# Cell-type–specific compartmentalization and function of the glucosinolate-myrosinase system in *Arabidopsis thaliana*

**DOI:** 10.1016/j.jbc.2026.113202

**Published:** 2026-05-27

**Authors:** Shweta Chhajed, Yatendra Singh, Hajra Maqsood, Craig Dufresne, Wenyuan Song, Sixue Chen

**Affiliations:** 1 Department of Biology, University of Florida, Gainesville, Florida, USA; 2 Department of Biology, University of Mississippi, Oxford, Mississippi, USA; 3 Division of Chromatography, Thermo Scientific Training Institute, West Palm Beach, Florida, USA; 4 Department of Plant Pathology, IFAS, University of Florida, Gainesville, Florida, USA; 5 Plant Molecular and Cellular Biology, University of Florida, Gainesville, Florida, USA; 6 Genetics Institute, University of Florida, Gainesville, Florida, USA

**Keywords:** glucosinolates, *Arabidopsis thaliana*, glucosinolate-myrosinase system, guard and mesophyll cells, selected reaction monitoring

## Abstract

Plants deploy chemical defense strategies to respond to abiotic and biotic stresses, including the glucosinolate-myrosinase (GM) system (“mustard oil bomb”) characteristic of *Brassicales*. Glucosinolates (GLSs) are specialized metabolites that, upon hydrolysis by myrosinases, generate bioactive products that deter herbivores and pathogens. While the biochemical functions of GLS breakdown products are well studied, the cell-type–specific organization of the GM system and its relevance to stomatal immunity remain incompletely defined. Here, we compare cell-type–enriched GLS profiles and myrosinase activities in guard cells (GCs) and mesophyll cells (MCs) of *Arabidopsis thaliana*. Using untargeted metabolomics, we identified 20 GLSs across seeds, leaves, GCs, and MCs with high mass accuracy, followed by targeted quantitative analysis of GLSs based on a selected reaction monitoring approach. Comparative analyses revealed pronounced differences in GLS composition between GCs and MCs, accompanied by distinct myrosinase activity profiles. Together, these findings establish a cell-type–specific model for GLS metabolism in *A. thaliana* and demonstrate differential organization of the GM system between GCs and MCs. This work provides foundational evidence linking the spatial organization of GLS metabolism to stomatal immune function and will springboard future mechanistic studies of the GM system in plant defense signaling.

Glucosinolates (GLSs) are sulfur-containing specialized metabolites characterized by a thioglucose group, a sulfonated oxime moiety, and a variable aglycone side chain (R group). They are produced by plants in the order of *Brassicales*, including many Brassicaceae vegetables and the reference species *Arabidopsis thaliana* (1, 2). GLSs can be categorized as aliphatic, indole, and aromatic based on the variable R groups, which are derived from different amino acids (3, 4, 5). Different precursor amino acids, variation in side-chain length caused by chain elongation, and extensive side-chain modifications lead to the chemical diversity of GLSs. There are more than 120 structurally distinct GLSs that differ from each other by the R group (6). Upon tissue disruption, GLSs are enzymatically hydrolyzed to produce a variety of biologically active compounds. The biological activities of GLS degradation products in plant defense against pathogens and herbivores have been well studied (7, 8). Several GLS-derived compounds, such as sulforaphane and indole-3-carbinol, have also been extensively studied for their roles in human health and disease prevention (9, 10).

In *A. thaliana*, GLS profiles vary across tissues and throughout developmental stages (11, 12, 13). Nearly 40 different GLSs with mostly indolic or aliphatic side chains have been identified from different *A. thaliana* ecotypes, demonstrating the vast chemical diversity (14). For instance, seeds are a sink for GLS accumulation during *A. thaliana* development (15). Aliphatic GLSs are predominantly abundant in leaves, whereas indole GLSs mainly accumulate in the roots of *A. thaliana* (16). Over the past three decades, the genes and pathways responsible for the biosynthesis of different GLSs have been well established (17), and vast genetic resources are now available. *A. thaliana cyp79b2/cyp79b3* double mutant produced aliphatic GLSs, which were derived from chain-elongated methionines (17), whereas *myb28/myb29* double mutant produces indolic GLSs derived from tryptophan (18, 19).

The compartmentalization of the GLS-myrosinase (GM) system (commonly referred to as the “mustard oil bomb”) is the spatial separation of GLSs and their hydrolyzing enzymes, myrosinases (β-thioglucosidases). The activation of the “mustard oil bomb” is typically triggered by physical damage. GLSs are hydrolyzed by myrosinases, resulting in various hydrolytic products, such as nitriles, thiocyanates, and isothiocyanates (ITCs) (20, 21). ITCs exhibit strong antimicrobial activity against a wide range of pathogens (22). Several other degradation products are involved in plant nutrition (23) and growth regulation (24). The efficacy of the “mustard oil bomb” highlights its importance in plant natural adaptation to abiotic and biotic stresses (22). Also, it is reasonable to hypothesize that the GLS degradation products may function as signaling molecules that regulate different biological processes, such as stomatal immune responses. Given the rapid kinetics of stomatal responses, a pre-existing, cell-autonomous GM system in the guard cells (GCs) may provide a mechanistic avenue for fast immune signaling against pathogen invasion.

To date, several studies have suggested that the GM system may be differentially localized in S-cells (25), GCs (26), myrosin cells (27), mesophyll cells (MCs), epidermal cells, trichomes, and vascular tissues (28). For example, GLSs are enriched in S-cells found in *A. thaliana* flower stalks and in the vicinity of myrosin cells enriched with myrosinases (29). These observations suggest that GLSs and myrosinases are compartmentalized in different cell types. The spatial distribution of GLSs was demonstrated in *A. thaliana* leaves by constructing ion intensity maps from MALDI-TOF-MS, where major GLSs were found to be more abundant in tissues of the midvein and the periphery of the leaf than in the inner lamina (30). Although this study concluded that GLSs are not abundant on *A. thaliana* leaf surfaces, the authors could not obtain information about the cell-type distribution of GLSs in leaves. Previous reports have shown altered GLS metabolism in GCs in response to abiotic stress induced by CO_2_ and abscisic acid (31, 32). These studies and others (25, 33, 34) seem to suggest that GLSs and myrosinases may co-exist in the same cells. While spatial separation of GLSs and myrosinases at the tissue and cell-type level is well supported, the extent of their cell-type–specific colocalization, dynamic regulation, and functional implication, particularly in specialized cells such as GCs, remains incompletely resolved (20, 35, 36).

Certain foliar bacterial pathogens, including *Pseudomonas syringae*, enter the plant tissue through stomatal pores. The GCs act as the first line of host defense to actively recognize the invasion and close the stomata as an innate immune response (37). GLSs coexist with myrosinases, which play a crucial role in defending plants against pathogens (28, 38). For example, overexpression of a myrosinase gene in *A. thaliana* accelerated stomatal closure and inhibited stomatal reopening upon bacterial *Pst* DC3000 infection (39). In addition, GCs were found to be enriched in myrosinases (40, 41). However, whether and how cell-type–specific distribution of GLSs and myrosinases contributes to these responses remains unresolved.

In this study, we aim to investigate the cell-type–specific organization of the GM system in GCs and MCs of *A. thaliana*. Using untargeted and targeted mass spectrometry approaches, we compare GLS profiles and myrosinase activities between these two functionally distinct cell types under identical physiological conditions. This work aims to provide a quantitative, cell-type–resolved framework toward understanding how differential organization of the GM system may relate to stomatal immune responses and pathogen defense.

## Results and Discussion

### Purity assessment of enriched guard cells and mesophyll cells

Effective isolation and verification of cell-type enrichment are prerequisites for interpreting cell-type–specific molecular and physiological differences. GCs and MCs were isolated from the same *A*. *thaliana* rosette leaves to enable direct comparison under identical physiological conditions. Cell isolation was performed using a previously described protocol (42). Fluorescein diacetate (FDA) and neutral red staining demonstrated that isolated GCs and MCs were intact and viable both before and after enzymatic treatment (Fig. S1*A*), consistent with previous reports (42, 43, 44). Chlorophyll measurements further supported the cell-type enrichment, as leaves and MCs displayed significantly higher chlorophyll a and b levels than GCs (Fig. S1*B*), reflecting the limited photosynthetic capacity of GCs (43).

Molecular validation using qRT-PCR revealed strong enrichment of GC marker genes *SLAC1* and *OST1* in GC samples compared to MCs. Conversely, photosynthesis-associated genes *PSBR* and *LHCA2* were significantly enriched in MCs (Fig. S1*C*). Consistent with previous transcriptomic studies, plasma membrane H^+^-ATPase genes *AHA6* and *AHA9* also showed preferential expression in GCs (Fig. S1*D*) (41, 45). Collectively, these independent physiological and molecular analyses support robust and reproducible enrichment of GCs and MCs. Having established robust enrichment and viability of GC and MC samples, we next examined GLS composition in each cell type.

### Identification of GLSs

**Figure 1:**
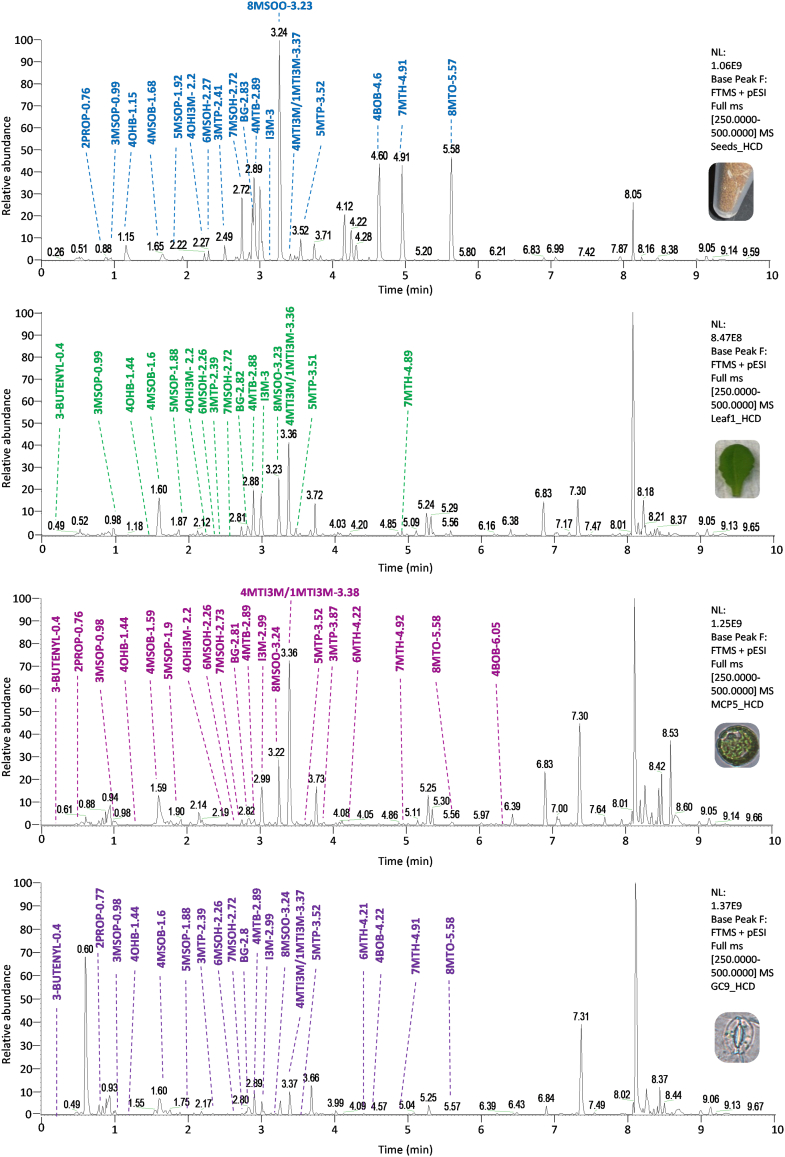
**Distinct glucosinolate profiles in *Arabidopsis thaliana* seeds, leaves, mesophyll cells (MCs), and guard cells (GCs).** The base peak chromatograms were acquired on the UHPLC-Orbitrap Fusion MS system in positive mode with accurate mass and high resolution for both MS^1^ and MS^2^.

**Figure 2:**
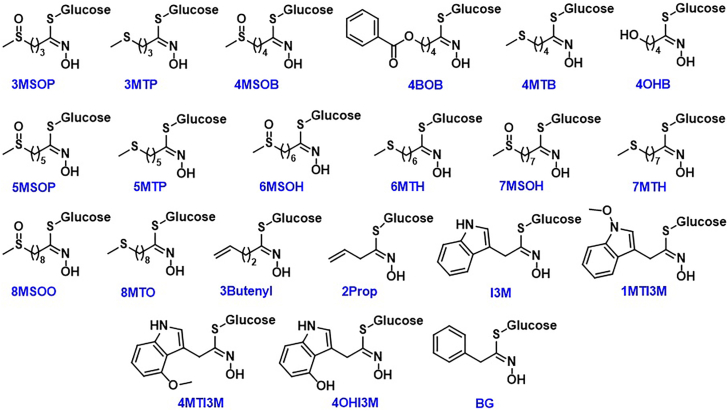
**Identified desulfo-GLS structures from seeds, leaves, guard cells, and mesophyll cells of *Arabidopsis thaliana*.** desulfo-GLS, desulfonated glucosinolate; 3MSOP, 3-(methylsulfinyl)-propyl; 3MTP, 3-(methylthio)-propl; 4MSOB, 4-(methylsulfinyl)-butyl; 4BOB, 4-benxoyloxybutyl; 4MTB, 4-(methylthio)-3-butenyl; 4OHB, 4-hydroxybutyl; 5MSOP, 5-(methylsulfinyl)-pentyl; 5MTP, 5-(methylthio)-pentyl; 6MSOH, 6-(methylsulfinyl)-hexyl; 6MTH, 6-(methylthio)-hexyl; 7MSOH, 7-(methylsulfinyl)-heptyl; 7MTH, 7-(methylthio)-heptyl; 8MSOO, 8-(methylsulfinyl)-octyl; 8MTO, 8-(methylthio)-octyl; 3Butenyl, 3-butenylglucosinolate; 2Prop, 2-propenyl glucosinolate; 4OHI3M, 4-hydroxy-indol-3-ylmethylglucosinolate; I3M, indol-3-ylmethyl; 1MTI3M, N-methoxy-indol-3-ylmethyl; 4MTI3M, 4-methoxy-indol-3-ylmethyl; BG, benzylglucosinolate.

Desulfo-GLSs extracted from seeds, leaves, GCs, and MCs were analyzed using a UHPLC-Orbitrap Tribrid MS using electrospray ionization (ESI) in positive mode. Across all sample types, a total of 20 GLSs were confidently annotated following level 2 metabolite identification of GLSs (46). Across all the samples, these peaks have similar retention time (Rt) and are eluted in the same order (Fig. 1), indicating little matrix interference (46). Mass spectrometry data of identified desulfo-GLSs that include chemical formula, accurate mass measurement, and MS/MS fragments are summarized in Table 1. The accurate mass was determined based on adducts [M + H]^+^ and/or [M+Na]^+^ formed in ESI positive mode, and the precursors were also confirmed using isotopic fidelity. Overall, the mass error < 3.00 ppm was considered, except for 4BOB (−3.65 ppm) (Table 1), enhancing the confidence in desulfo-GLS identification. The characteristic fragment ions, [M + H]^+^ and [M+Na]^+^, of each GLS were identified and analyzed using Mass Frontier 8.0. With the assistance of this software, the MS/MS spectra were annotated with structural information (Fig. S2). Based on the criteria described in the LC-MS/MS analysis section, a total of 20 GLSs were unambiguously identified from seeds, leaves, GCs, and MCs of *A. thaliana* (Table 1, Figs. 1 and 2).

**Table 1:** Mass spectrometry data of the identified desulfo-GLSs in the seed, leaves, guard cells, and mesophyll cells of *Arabidopsis thaliana*

GLSs	Intact mass	Chemical formula	Desulfo-GLS Exp. *m/z*	Desulfo-GLS Cal. *m/z*	Error (ppm)	Desulfo-GLS Neutral mass	(+) MS/MS *m/z* of major diagnostic fragment ions for desulfo-GLSs
3MSOP	423.0328	C_11_H_21_NO_7_S_2_	366.0653 [M+Na]^+^	366.0652	0.27	343.0759	343.6844, 219.0309, 204.0127, 185.0419, 170.0424
3MTP	407.0378	C_11_H_21_NO_6_S_2_	350.0702 [M+Na]^+^	350.0702	0.00	327.0810	219.0308, 188.0360, 185.0360
4MSOB	437.0484	C_12_H_23_NO_7_S_2_	380.0810 [M+Na]^+^	380.0808	0.52	357.0916	218.0284, 200.0173, 185.0421
4BOB	495.0869	C_18_H_25_NO_8_S	438.1193 [M+Na]^+^	438.1193	−3.65	415.1301	276.0665, 242.0791, 185.0420
4MTB	435.0328	C_12_H_23_NO_6_S_2_	364.0864 [M+Na]^+^	364.0859	1.37	341.0967	219.0308, 202.0340, 185.0418, 168.0457
4OHB	377.0450	C_11_H_21_NO_7_S	334.0931 [M+Na]^+^	334.0928	0.89	311.1039	219.0290, 185.0420, 172.0407
5MSOP	451.0641	C_13_H_25_NO_7_S_2_	372.1149 [M+H]^+^	372.1145	1.07	371.1072	210.0624, 192.0515, 177.0407
5MTP	434.0619	C_13_H_25_NO_6_S_2_	378.1020 [M+Na]^+^	378.1016	1.05	355.1123	219.0309, 216.0133, 185.0419
6MSOH	464.0724	C_14_H_27_NO_7_S_2_	408.1132 [M+Na]^+^	408.1121	2.69	385.1229	246.0606, 219.0294, 212.0726, 185.0421
6MTH	449.0842	C_14_H_27_NO_6_S_2_	392.1174 [M+Na]^+^	392.1172	0.51	369.1280	230.0647, 219.0308, 196.0776, 185.0419
7MSOH	479.0954	C_15_H_29_NO_7_S_2_	422.1287 [M+Na]^+^	422.1278	2.13	399.1385	260.0751, 242.0649, 219.0294, 185.0421
7MTH	462.0932	C_15_H_29_NO_6_S_2_	406.1333 [M+Na]^+^	406.1329	0.98	383.1436	244.0309, 219.0309, 210.0920, 185.0420
8MSOO	493.1110	C_16_H_31_NO_7_S_2_	436.1442 [M+H]^+^	436. 1434	1.83	413.1542	274.8960, 256.0796, 240.1034
8MTO	476.1088	C_16_H_31_NO_6_S_2_	420.1492 [M+Na]^+^	420.1485	1.66	397.1593	258.0959, 224.1089, 219.0309, 185.0420
3-butenyl	373.0501	C_11_H_19_NO_6_S	316.0824 [M+Na]^+^	316.0825	−0.31	293.0933	298.2753, 280.2636, 219.0309, 185.0419, 154.0306
2-prop	396.9903	C_10_H_17_NO_6_S	302.3062 [M+Na]^+^	302.3069	−2.31	279.0777	284.2942, 219.0292, 185.0419, 140.0136
4OHI3M	463.0481	C_17_H_22_N_2_O_8_S	407.0884 [M+Na]^+^	407.0883	0.24	384.4030	245.0360, 219.0292, 211.0484, 185.0419, 168.0423, 146.0600
I3M	448.0610	C_16_H_20_N_2_O_6_S	369.1117 [M+H]^+^	369.1115	0.54	368.1042	249.0698, 207.0595, 174.0374, 130.653
1MTI3M	478.0716	C_17_H_22_N_2_O_7_S	399.1220 [M+H]^+^	399.1220	0.50	398.1148	263.0595, 237.1176, 219.1078, 160.0756
4MTI3M	478.0716	C_17_H_22_N_2_O_7_S	399.1222 [M+H]^+^	399.1220	0.50	398.1148	263.0595, 237.1176, 219.1078, 160.0756
BG	409.0501	C_14_H_19_NO_6_S	352.0829 [M+Na]^+^	352.0825	1.13	329.0933	219.0309, 190.0299, 185.0419

GLS, glucosinolate; desulfo-GLSs, desulfonated glucosinolates; Exp., experimental; Cal., calculated; MW, molecular weight; *m/z*, mass to charge ratio; 3MSOP, 3-(methylsulfinyl)-propyl; 3MTP, 3-(methylthio)-propl; 4MSOB, 4-(methylsulfinyl)-butyl; 4BOB, 4-benxoyloxybutyl; 4MTB, 4-(methylthio)-3-butenyl; 4OHB, 4-hydroxybutyl; 5MSOP, 5-(methylsulfinyl)-pentyl; 5MTP, 5-(methylthio)-pentyl; 6MSOH, 6-(methylsulfinyl)-hexyl; 6MTH, 6-(methylthio)-hexyl; 7MSOH, 7-(methylsulfinyl)-heptyl; 7MTH, 7-(methylthio)-heptyl; 8MSOO, 8-(methylsulfinyl)-octyl; 8MTO, 8-(methylthio)-octyl; 3Butenyl, 3-butenylglucosinolate; 2Prop, 2-propenyl glucosinolate; 4OHI3M, 4-hydroxy-indol-3-ylmethylglucosinolate; I3M, indol-3-ylmethyl; 1MTI3M, N-methoxy-indol-3-ylmethyl; 4MTI3M, 4-methoxy-indol-3-ylmethyl; BG, benzylglucosinolate.

**Figure 3:**
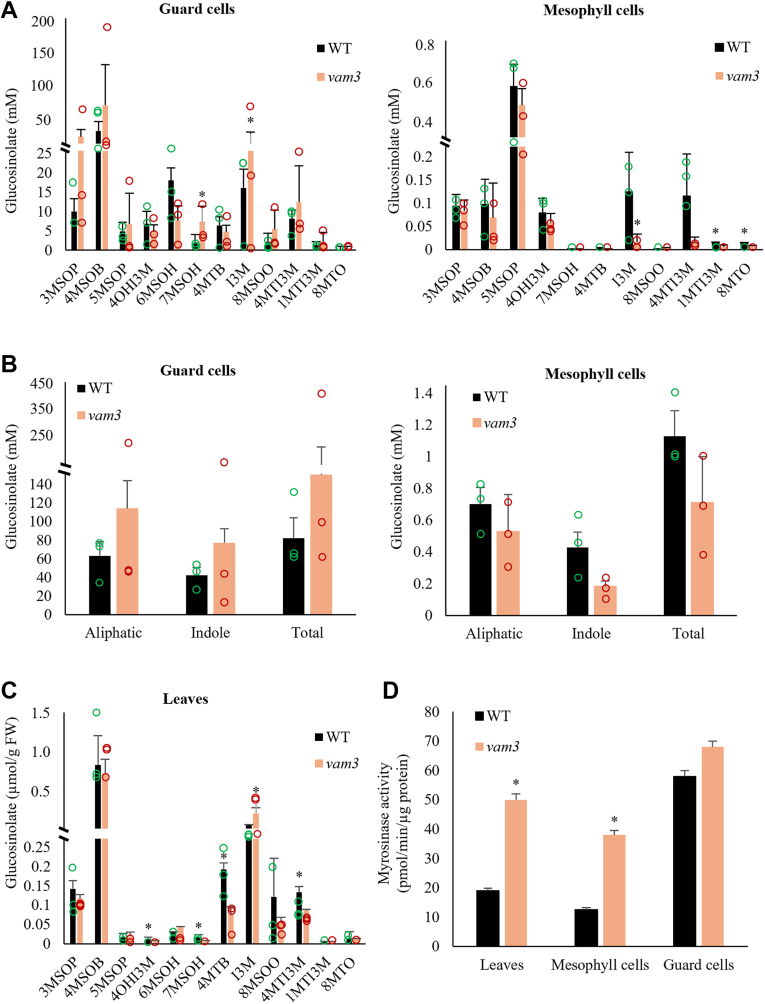
**Glucosinolate (GLS) profiles and myrosinase activities in different cell types of *Arabidopsis thaliana*.***A*, quantitative GLS profiles in guard cells (GCs) and mesophyll cells (MCs) of WT and *vam3*, showing GLS concentrations on a per-cell basis; (*B*) aliphatic, indolic, and total GLS concentrations in GCs and MCs of WT and *vam3*; (*C*) glucosinolate concentrations in leaves of WT and *vam3*; (*D*) myrosinase activities in leaves, MCs, and GCs of WT and *vam3*. Data show mean (±SE) from three biological replicates (*p* < 0.05), with individual points as a scatter plot. Pairwise *t* test was conducted, and ∗ indicates significant difference between the two genotypes.

The aliphatic GLSs are derived from different chain-elongated methionines (17). The elongation process results in various side-chain lengths of GLSs that are pivotal for plant defense mechanisms. Using high-resolution mass spectrometry, fifteen GLSs were identified as aliphatic GLSs, that is, 3MSOP, 3MTP, 4MSOB, 4MTB, 4OHB, 5MSOP, 5MTP, 6MSOH, 6MTH, 7MSOH, 7MTH, 8MSOO, 8MTO, 3-butenyl, and 2-prop, which are carried from three to eight methylene groups (Fig. 3). Here specific fragment ions for aliphatic desulfo-GLSs showed a neutral loss of anhydrous glucose (anhydroGlc, C_6_H_10_O_5_, 162 Da) and yielded fragment ion at *m/z* 204.0127 for 3MSOP (Fig. S2*A*), *m/z* 188.036 for 3MTP (Fig. S2*B*), *m/z* 218.0284 for 4MSOB (Fig. S2*C*), *m/z* 202.034 for 4MTB (Fig. S2*E*), *m/z* 172.0407 for 4OHB (Fig. S2*F*), *m/z* 210.0624 for 5MSOP (Fig. S2*G*), *m/z* 216.0133 for 5MTP (Fig. S2*H*), *m/z* 246.0606 for 6MSOH (Fig. S2*I*), *m/z* 230.0647 for 6MTH (Fig. S2*J*), *m/z* 260.0751 for 7MSOH (Fig. S2*K*), *m/z* 244.0309 for 7MTH (Fig. S2*L*), *m/z* 274.896 for 8MSOO (Fig. S2*M*), *m/z* 258.0959 for 8MTO (Fig. S2*N*), *m/z* 154.0306 for 3Butenyl (Fig. S2*O*), and *m/z* 140.0136 for 2Prop (Fig. S2*P*). These product ions result from the cleavage of the thioglucosidic bond connecting the sugar moiety to the sulfur group of the thiol, thus confirming the GLS structures (47, 48).

Aromatic GLSs are derived from phenylalanine and tyrosine. The 4BOB was identified as aromatic desulfo-GLSs (Table 1, Figs. 2 and S2, *D*, *U*). Four GLSs derived from tryptophan (49), 4OHI3M, I3M, 1MTI3M, and 4MTI3M were identified as indolic GLSs (Fig. 3). I3M yields a distinctive ion at *m/z* 130.6530 equivalent to [Indole-CH_2_]^+^ (Fig. S2*R*). Similarly, 4OHI3M generates a fragment ion at *m/z* 146.0600 corresponding to [4-hydroxyindole-CH_2_]^+^ (Fig. S2*Q*). The ions produced by the positional isomers also revealed that *m/z* 160.0756 is linked to [1-methoxyindole-CH_2_]^+^ from 1MTI3M and [4-methoxyindole-CH_2_]^+^ from 4MTI3M (Fig. S2, *S* and *T*). 1MTI3M and 4MTI3M co-eluted at Rt. 3.36 min (Fig. 1), sharing the same molecular weight and chemical formula (Table 1). While their MS/MS spectra are similar, they differ in the intensities of fragment ions (Fig. S2, *S* and *T*). These GLSs are identified as positional isomers. In 1MTI3M, the methoxy is linked to the nitrogen atom of indole, but in 4MTI3M, the methoxy is present at a fourth carbon of benzene (Fig. 3). Careful analysis of the Orbitrap product ions provides insight into the structural identities of these indolic GLSs.

Interestingly, most of the [desulfo-GLSs-Na]^+^ adducts fragmented yield [thioGlc + Na]^+^ product ion at *m/z* 219.0308 by neutral loss of the indolic or aliphatic chain, while fragmentation at another side of the S was also observed, leading to [anhydroGlc + Na]^+^ product ion at *m/z* 185.0420 produced by loss of the indolic or aliphatic chain containing the thiol group, which is consistent with existing literature (47, 50, 51). Subsequently, these fragment ions provided clues to confirm the unknowns belonging to the GLS family further by selecting “Fragment Ion Search” detection on the Mass Frontier.

Cell-type–specific analysis revealed distinct GLS distributions that are masked in whole-leaf measurements. Consistent with previous reports, 6MTH and 3-butenyl GLSs were absent from seeds (11), while 4BOB, 8MTO, and 2-propyl GLSs were not detected in leaves (Fig. 1). All annotated GLSs were detected in MCs, consistent with their proposed role as a major site of GLS biosynthesis (27). In contrast, GCs exhibited a more selective GLS profile, with 4OHI3M notably absent and pronounced differences in relative abundance compared to MCs (Fig. 1). These results indicate cell-type–specific GLS enrichment and suggest functional specialization of the GM system in GCs.

### Glucosinolate profiles in MCs and GCs

GLS quantification has been done for decades using the standard FDA-approved LC-UV method with known response factors for different GLSs (52). GLS standards have limited availability and are expensive. Therefore, the strategy adopted for this study involved annotating GLSs using a well-established *A. thaliana* seed GLS profile (53, 54) with high confidence (*e.g.*, error in ppm < ±3 and MS^2^ spectra).

The identified 20 GLSs with accurate mass precursor and product ions were profiled in different GCs and MCs using targeted UPLC-TSQ Altis MS. *A. thaliana* seeds are known to accumulate GLSs as sinks (12); therefore, the seed GLSs were used to optimize the SRM transitions (Table S2). Following the transition optimization, cell-type–specific profiling of GC and MC samples was recorded (Figs. 3*A* and S3). Aliphatic GLSs (4MSOB, 4MTB, and 8MSOO) and indolic GLSs (I3M, 4MTI3M, 1MTI3M, and 4OHI3M) are present in both types of cells, but there are significant differences in the GLS profiles between the two cell types. GCs seem to have 100 times more GLS concentration than MCs (Fig. 3*A*). Aliphatic 4MSOB was found to be in the highest concentration in the GCs, whereas indolic 4MTI3M and aliphatic 4MSOB were relatively high in the MC profile. Overall, total aliphatic GLSs were found to be more than indolic GLSs in GCs and MCs (Fig. 3*B*). Altogether, these findings support the notion that GLSs display spatial organization in different cell types. Here, GCs have been revealed to be a “hot spot” of GLSs. Although this system has been studied for decades in whole leaves against various biotic and abiotic stresses, its contents in individual single-cell types are far from complete. Studies conducted by Koroleva *et al.* showed that GLSs are particularly high in S-cells (mM range) based on sulfur distribution (25, 29). S-cells, however, were not directly analyzed for GLS profiles and myrosinases. The single cell-based trichomes contain GLSs, and their degradation products like ITCs accumulate to higher levels in trichomes than in other parts of the leaves (28). Cumulatively, these efforts to comprehend the cell-type–specific GLS contents have led to interesting questions on the cellular/spatial organization of the GM system. Our study provides strong evidence for the differential organization of the GLSs in GCs and MCs of *A. thaliana*.

Many *A. thaliana* mutants in the GM system are available. For example, the *cyp79b2cyp79b3* double mutant lacks indolic GLSs in the leaves and trichomes, and aliphatic GLS levels remain unaltered (28). A *vam3* mutant (deficient in the regulation of vesicular transport of vacuolar proteins) accumulates thioglucoside glucohydrolases 1 and 2 (TGG, also called myrosinases) in its leaves (55). It may be reasonable to hypothesize that the *vam3* leaves, GCs, and MCs have lower GLS levels than WT. GLS levels of *vam3* and WT were measured to test this hypothesis. As shown in Figure 3*C*, two aliphatic GLSs (7MSOH and 4MTB) and two indolic GLSs (4OHI3M and 4MTI3M) were significantly higher in WT leaves than in the *vam3* leaves. In contrast, two aliphatic 5MSOP and 8MTO in the *vam3* leaves were higher than in the WT leaves. Interestingly, no significant differences in these GLSs were found in the *tgg1/tgg2* double mutant compared to the WT (56). In WT GCs, only two aliphatic GLSs (4MTB and 6MSOH) were slightly higher, though not significantly. In contrast, several GLSs, including 3MSOP, 4MSOB, 5MSOP, 7MSOH, 8MSOO, I3M, 4MTI3M, and 1MTI3M, showed higher levels in the *vam3* GCs. In WT MCs, 8MTO, I3M, and 1MTI3M levels were higher than those in *vam3* MCs (Fig. 3*A*). Clearly, highly abundant myrosinases in the *vam3* mutant do not necessarily cause decreases in different GLSs (Fig. 3, *A*–*C*).

To test whether myrosinase activities correlate with the GLS profiles, myrosinase activities in leaves, GCs, and MCs were measured. As shown in Figure 3*D*, GCs exhibited the highest activities, whereas MCs showed the lowest activities. This activity result correlates well with the high abundance distribution of myrosinases in GCs (57, 58, 59), but it does not explain the high levels of GLSs in the GCs if *in vivo* degradation is controlled by the myrosinase activities. The confirmed colocalization of GLSs and myrosinases within the same cell types and subcellular compartments (vacuole) (34) is supported by 2D gel maps of vacuolar proteins from *A. thaliana* rosette leaves, which demonstrate the presence of myrosinases (*TGG1*, *TGG2*, and myrosinase-associated protein (MyAP)) in vacuoles, thereby providing proteomic evidence for their subcellular localization (Fig. S4) (60). The regulation of myrosinase activity is probably affected by subcellular environment, such as pH, ligands, interacting proteins, and possibly posttranslational modifications essential for its functions. However, the precise molecular mechanism remains elusive and necessitates additional research to elucidate how the vacuolar microenvironment affects GLS metabolism under different conditions, for example, during immune responses.

### The role of the GM system in stomatal movement

GCs are unique in containing high concentrations of GLSs (Fig. 3, *A* and *B*) and abundant myrosinases (Fig. 3*D*) (26, 32). To understand the role of the GM system in GC functions, we took the reverse genetics approach in analyzing the stomatal movement phenotype. A quadruple mutant (*myb28/29cyp79b2/b3*) defective in producing both aliphatic and indolic GLSs (17, 18, 19) showed a significantly smaller stomatal aperture index than the WT (Fig. S5*A*). The aliphatic GLS mutant *myb28/29* and the indolic GLS mutant *cyp79b2/b3* also had significantly smaller stomatal apertures than the WT, but larger than the quadruple mutant.

As to myrosinases, the double mutant *tgg1/tgg2* and the over-expression mutant (*vam3*) did not differ significantly in stomatal aperture index (Fig. S5*A*). The stomatal density of WT is lower than that of *vam3*, but the stomatal aperture in WT is larger than that in the mutant (Fig. S5, *A* and *B*). The GM system is a well-studied defense mechanism in Brassicales, including *A. thaliana* and several other crops. It has been shown to promote stomatal closure in response to pathogen invasion, serving as a crucial component of plant innate immunity (23, 61). For example, overexpression of a myrosinase gene, *BoTGG1* from broccoli (*Brassica oleracea* var. italica) enhanced stomatal defense against *Pst DC3000* and delayed flowering in *A. thaliana* (39). The functional diversity of GLS hydrolysis products, such as ITCs, plays a significant role in plant defense against different pathogens and pests (62). These results indicate that perturbation in the GM systems affects stomatal movement. Also, the absence of myrosinase was shown to affect stomata, cuticles, and cell walls that are known as defense barriers (59, 63). The next obvious question is how relevant *in vivo* GLS turnover is to stomatal defense or apoplastic defense.

**Figure 4:**
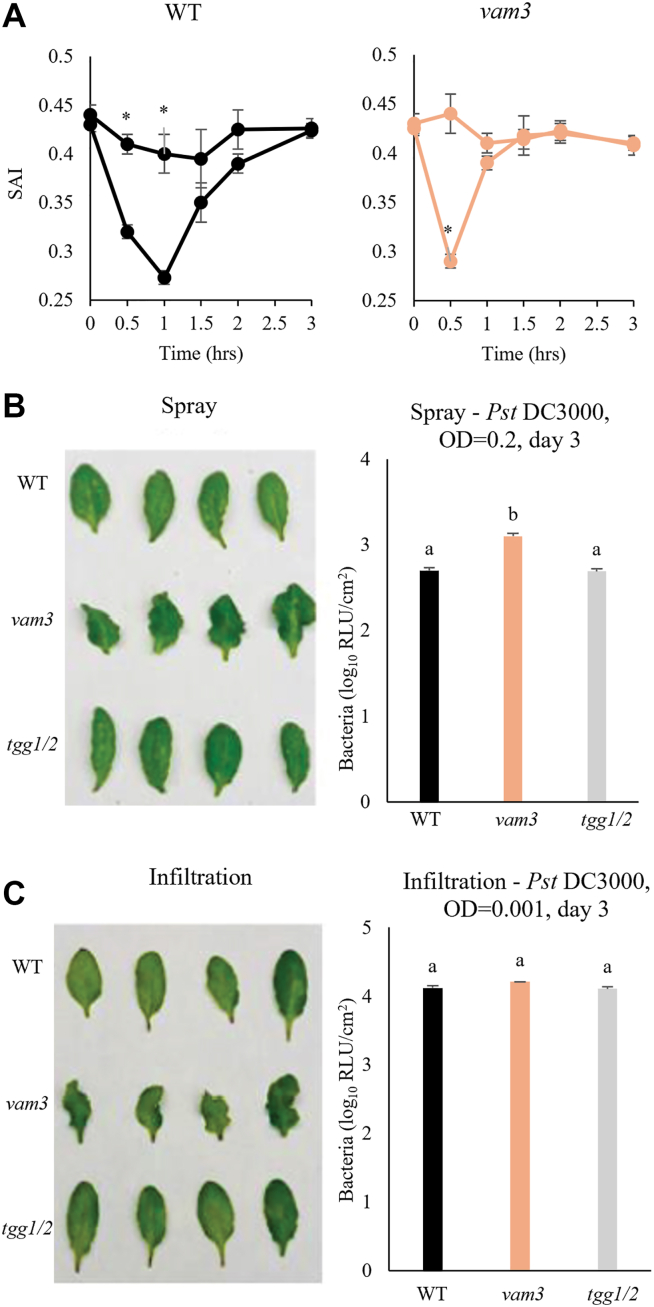
**Stomatal immunity and apoplastic defense assessment of WT, *vam3*, and *tgg1/tgg2* mutants.***A*, *orange* line denotes the stomatal movement (indicated by the stomatal aperture index (SAI)) in response to spray treatment of *Pst DC3000* (OD600 = 0.2), whereas the *blue* line represents mock treatment of the WT and *vam3*. Images of mock peels and treatment peels were taken at 0, 0.5, 1, 1.5, 2, and 3 h. Pairwise *t* test was conducted, and ∗ indicates significant difference in SAI. *B*, leaf phenotype and bacteria growth assay after spray (OD600 = 0.2) or (*C*) infiltration (OD600 = 0.001) of *Pst DC3000*. Both the spray results and the infiltration results were obtained at day 3 after inoculation. Data represented mean (±SE) from eight independent biological replicates, and different letters indicate statistical significance (*p* < 0.05; Anova test). RLU, relative luminescence unit.

The phenotype of *A. thaliana* stomatal response to *Pst* DC3000 has been studied since 2006, where the stomata close at 1 h and reopen at 3 h post-treatment (Fig. 4*A*) (64, 65). Interestingly, the *vam3* mutant exhibited earlier stomatal closure and reopened the stomata earlier than the WT (Fig. 4*A*). This indicates that the GCs' response to the pathogen attack in *vam3* was hypersensitive compared to the WT, that is, the ignition of the “mustard oil bomb” might be accelerated in *vam3,* possibly due to increased availability of myrosinases and GLS substrates. Thus, we hypothesize that myrosinases play an active role in pathogen-triggered GLS degradation. Indeed, myrosinase activity in the *vam3* mutant was found to be significantly higher than in WT leaves. A similar trend was followed in GCs and MCs (Fig. 3*D*). Unsurprisingly, the GCs showed higher activity than leaves and MCs. The high levels of GLSs in GCs may account for the higher myrosinase activity and stomatal functionality (Fig. 3). The higher GC myrosinase activity was also confirmed by an orthogonal assay (Fig. S6). Moreover, the expression levels (heat map), tissue localization, and accumulation of myrosinase (TGG1/TGG2), myrosinase-binding proteins, and myrosinase-associated protein were retrieved from the E-plant (https://bar.utoronto.ca/eplant/) database and heatmapper (http://www.heatmapper.ca/). Clearly, *TGG1*, *TGG2*, and *MyAP1* are highly expressed in the GCs as compared to MCs (Fig. S7).

Functional characterization using a spray-based bacterial assay revealed that the *vam3* mutant exhibited noticeably higher bacterial growth after bacterial spray (Fig. 4*B*), suggesting that the mutant is more susceptible. No significant differences in bacterial growth were observed between WT and mutant plants in the infiltration experiment (Fig. 4*C*). The *tgg1*/*tgg2* double mutant shows the absence of a significant phenotype during the stomatal immunity assay. This indicates that other myrosinase-related enzymes may play a role, highlighting the functional redundancy and complexity of the GM system. For instance, PEN2 (Penetration 2) is an atypical myrosinase that promotes the hydrolysis of indole GLSs (66, 67). PEN2 may operate within a broader modular immune complex, capable of compensating for the lack of typical *TGG* activities following pathogen exposure. BGLU28 is another atypical myrosinase recently shown to facilitate GLS degradation while simultaneously modulating redox equilibrium, which is crucial for GC functionality (56). Given that stomatal immunity involves redox signaling and ion transport, it is logical that the activities of PEN2, BGLU28, or other β-thioglucosidases in the GCs could affect stomatal function and defensive mechanisms.

Several studies have been published on *A. thaliana* immune responses and bacterial infection (61, 64, 68). However, the treatment of *vam3* with bacteria has not been previously reported. As shown in Figure 4*A*, overexpression of *TGGs* in *vam3* may accelerate the “mustard oil bomb” reaction, resulting in a rapid stomatal closure, but a premature restoration of the stomatal opening allows pathogen entry. Hence, the spray-inoculated *vam3* plants exhibited more necrosis, chlorosis, and significantly greater bacterial growth relative to WT, indicating susceptibility through the stomatal pathway (Fig. 4*B*). When stomatal immunity is by-passed through infiltration, the bacterial growth difference between WT and *vam3* became minimal, suggesting that myrosinases may have a pivotal role in stomatal immunity and a limited contribution towards apoplastic immunity.

## Conclusion

Targeted quantification and myrosinase activity assays revealed differential compartmentation of the GM system in GCs and MCs. GCs exhibited higher GLS content and elevated myrosinase activities compared to MCs and leaves. These observations suggest that the spatial distribution of the GM system may be associated with stomatal immunity, rather than apoplastic defense against *Pst* DC3000. The findings also point to potential internal regulatory mechanisms within the GM system that vary between cell types. The results form a working model for further hypothesis testing experiments. More investigations are needed to explore cellular and subcellular compartmentation changes (*e.g.*, myrosin cells *versus* GCs), the roles of myrosinase-interacting proteins, and GLS degradation products as signaling molecules in GCs and MCs to better understand their contribution to stomatal immunity and overall plant defense.

## Experimental procedures

### Plant materials

*A. thaliana* seedlings were germinated from seeds in a potting mixture (Sungro Horticulture Propagation Mix) in a growth chamber with a light intensity of 160 μmol photons m^−2^ s^−1^ and a short-day photoperiod of 8 h light at 22 °C and 16 h dark at 20 °C. They were fertilized once a week with fertilizer 20-10-20 peat lite per the manufacturer's instructions. Thirty-eight-day-old plants were used for GLS extractions. Four biological replicates were used for each cell type, and each replicate had 50 peels of enriched GCs or corresponding MCs (see below). Dr Georg Jander from the Boyce Thompson Institute kindly provided the *tgg1*/*tgg2* mutant, Dr Ikuko Hara-Nishimura from Kyoto University gifted the *vam3* mutant, and Dr Judy Brusslan from the California State University gifted the other GLS mutants.

### GC and MC protoplast preparation

Fully expanded leaves of the *A. thaliana* seedlings were sandwiched between two layers of clear scotch tape. With the help of tweezers, the scotch tape was pulled apart from each layer. GCs were recovered from the lighter side, while MCs were obtained from the darker side of the leaves (36, 69). The lighter part of the leaf taped peel with GCs was placed in an opening buffer (50 mM KCl, 10 mM MES, pH 6.2 with KOH) and kept under the light. Care was taken so that the peels did not overlap or stick to each other. Fifty lighter peels containing GCs were treated with 25 ml enzyme solution (0.7% Calbiochem cellulysin R10, 0.025% Macerozyme R10, 0.25% BSA, and Y23 pectinase in opening buffer) for 7 min on a shaker to remove epidermal and vascular cells. Following the digestion, the peels were rinsed with the opening buffer twice for 5 min each and placed under light for 1 h in the opening buffer to facilitate recovery after the enzyme shock. Then the enriched GCs were frozen in liquid nitrogen and stored at −80 °C.

MC protoplasts were isolated according to a previous method (42), with slight modifications. The darker leaf peels were exposed to 15 ml enzyme solution (0.5% (w/v) macerozyme R-10 (Yakult Honsha), 1% (w/v) Onozuka RS cellulose (Yakult Honsha), 0.01% (w/v) pectolyase Y-23 (Seishin Pharmaceutical), 0.2% (w/v) BSA (Sigma), 0.1% (w/v) PVP-40 (Sigma), 10 mM MES-KOH (pH 5.5), 0.6 M Sorbitol, 0.5 mM CaCl_2_, 0.5 mM MgCl_2_, and 10 μM KH_2_PO_4_) to release the protoplasts. The peels were subjected to digestion in the dark at room temperature for 3 h with gentle agitation. The suspension was passed through a nylon mesh with a pore size of 200 μm, and the filtrate was transferred to 15 ml centrifuge tubes, followed by centrifugation at 150×*g* for 10 min. The pellet was resuspended in 5 ml solution 1 (10 mM MES-KOH with pH 5.5 and 0.6 M sucrose). A 5 ml solution 2 (10 mM MES-KOH with pH 5.5, add 0.6 M sorbitol, 0.5 mM CaCl_2_, 0.5 mM MgCl_2_, and 10 μM KH_2_PO_4_) was carefully dispensed on top of solution 1. After centrifugation at 150×*g* for 7 min, the interface containing MCs was transferred to a new tube and centrifuged at 150×*g* for 3 min, and the final pellet was gently resuspended in 10 ml of solution 2. The solution was kept in the dark for 1 h and then centrifuged at 150×*g* for 5 min to pellet the cells. The MCs were promptly frozen in liquid nitrogen and stored at −80 °C till further analysis.

### Cell viability staining and chlorophyll assay

Staining for cell viability was performed using bright-field and fluorescent microscopy. The two staining chemicals were used to test cell viability. First, 10 μl of 3% neutral red was added to 100 μl of the cells, and images were captured using a bright-field microscope. Second, 10 μl of 1 mM FDA was added to the cells and incubated for 5 min. For FDA viability imaging, the filter was changed to excitation at 494 nm and emission at 521 nm (70).

Chlorophyll assay was performed using a previously reported procedure (71, 72) with minor modifications. Four fully expanded leaves were mashed with a pestle in 800 μl of 80% acetone in a 1.5 ml tube and placed in the dark for 30 min to extract chlorophyll. This extract was centrifuged at 15,000×*g* for 15 min to pellet cellular debris, and the supernatant was used for spectrophotometric analysis. A blank for leaves and MCs was prepared in 80% acetone, while a blank for GCs was the supernatant of scotch tape ground in 80% acetone. Absorbance was measured at 663 nm and 646 nm to measure chlorophyll *a* and *b*, respectively. Total chlorophyll *a* and *b* were determined using the following formulas: (12.7 × A_663_ - 2.69 × A_646_) × volume/weight = chlorophyll *a* in mg/g of fresh weight, whereas = (22.9 × A_646_ - 4.86 × A_663_) × volume/weight = chlorophyll *b* in mg/g of fresh weight (72).

### Total RNA extraction, cDNA synthesis, and RT-qPCR

For total RNA extraction, 100 mg of leaves, GCs, and MCs (prepared from 100 leaves) were used. Three biological replicates were ground in liquid nitrogen with a pestle and mortar. Total RNA was extracted using a Qiagen RNeasy Plant mini kit (Cat. No. 74904) with DNase treatment through on-column DNase digestion (RNase-Free DNase Set from NEB). After assessing RNA quality and integrity on a nanodrop, 1% agarose gel electrophoresis was run. A total of 500 ng RNA was used for cDNA synthesis with random hexamer primers in a 20 μl reaction from the NEB ProtoScript II First Strand cDNA synthesis kit (E6560S). Affymetrix USB VeriQuest SYBR Green qPCR Master mix with Fluorescein (2×) (Product number 75600) was used for RT-qPCR reaction and analysis in a CFX96 real-time system with a C1000 Touch thermal cycler from Bio-Rad. RT-qPCR of GC and MC marker genes was performed using the housekeeping gene *ACTIN2* according to the manufacturer's instructions. Relative expressions were calculated using the delta Ct method and normalized to *ACTIN2*. Coding sequences of GC marker genes slow anion channel-associated 1 (*SLAC1*) and open stomata 1 (*OST1*), whereas MC marker genes photosystem II subunit R (*PSBR*), photosystem I light harvesting complex protein (*LHCA2*), and housekeeping/reference gene were used for primer design. (Table S1).

### Desulfoglucosinolate extraction

GLSs were extracted according to previously reported methods (12, 17), with slight modifications. The seeds (20 mg), leaves (200 mg, fresh weight), GCs (72 peels), and MCs (72 peels) (four biological replicates each) were ground in liquid nitrogen. To the ground samples, 1 ml of hot (80 °C) 70% methanol was added along with 40 μl of 5 mM benzyl glucosinolate as an internal standard and shaken for 10 min at 80 °C using a thermomixer. After centrifugation at 12,000×*g* for 5 min at 4 °C, the supernatant was transferred to a new tube. The extraction with 1 ml hot methanol was repeated twice. The supernatant was combined, and the volume was adjusted to 4 ml with 70% methanol. The extracts from each sample were loaded into the DEAE Sephadex A-25 ion exchange column. The column was washed twice with 70% methanol, followed by 2 ml of water and 1 ml of 20 mM acetate buffer. Desulfation was achieved by adding 0.5 ml of 0.25% sulfatase solution in 20 mM acetate buffer to each column. The column was kept overnight at room temperature. The next day, elution of the desulfo-GLSs was performed using water (1.5 ml, twice), followed by lyophilization of the eluted fraction using a SpeedVac. The desulfo-GLSs were stored at −20 °C and reconstituted in 100 μl, 60 μl, and 50 μl of water for the seeds, leaves, GCs, and MCs, respectively, before LC-MS/MS analysis.

### LC-MS/MS analysis

The structural analysis of the GLSs was performed on UHPLC Vanquish Horizon (Thermo Fisher Scientific) interfaced with Orbitrap Fusion MS (Thermo Fisher Scientific). A Thermo Scientific Accucore Gold RP-MS column (2.6 μm, 2.1 × 100 mm) was used to separate 10 μl aliquots of each sample at a flow rate of 0.35 ml/min and a column temperature of 55 °C during a 10 min run. The gradient comprises 0.1% formic acid in water (solvent A) and 0.1% formic acid in 100% acetonitrile (solvent B). The gradient elution was applied from 0 to 50% B (0–7 min), 50 to 95% B (7–7.5 min), 95-95% B (7.5–8.2 min), 95-0% B (8.2–8.5 min), and 0 to 100% A (8.2–10 min). The eluant was introduced to the Orbitrap Fusion MS through an ESI source. The MS was operated in ESI positive mode with a spray voltage of 3000 V, sheath gas of 45 Arb, transfer capillary temperature of 320 °C, vaporizer temperature of 350 °C, and auxiliary gas of 8 Arb. The focusing RF lens was set at 55%. The Orbitrap MS scan resolution of 120K at 500 *m/z*, range 150 to 500 *m/z* was used, and the AGC target was set to 400,000, and the maximum inject time was 50 ms. MS/MS spectra were acquired using a higher-energy collisional dissociation at a cycle time of 6 with a collision energy of 22%. Fragment ions were detected using Orbitrap image current. The AGC accumulation target was set to 200,000, the maximum injection time was set to 22 ms, and the resolution was set at 15,000. Thermo Scientific Xcalibur 4.6 software was used for data processing and analysis.

To achieve level 2 (46) metabolite identification of the desulfo-GLSs, a four-step analysis strategy was applied: (1) MS/MS accurate mass spectra were searched in Thermo Mass Frontier 8.0 software for spectral matching and annotation, and unidentified fragments from Mass Frontier 8.0 were annotated; (2) theoretical isotopic abundance matched with experimental abundance up to the 4th decimal points with mass accuracy of < 3 ppm; (3) isotopic fidelity confirms molecular formulas with high confidence; and (4) whenever possible, both H^+^ and Na^+^ adducts were considered. This strategy provides confident structural identification of the desulfo-GLSs.

Targeted quantitative analysis was performed on the same UHPLC Vanquish Horizon coupled to a Thermo Scientific TSQ-ALTIS-MS equipped with an ESI source operated in positive mode using the above chromatographic method. The internal standard (benzyl glucosinolate) was used to optimize the RF lens and collision energies on the TSQ-ALTIS-MS. Since GLS standards are not readily available, seed GLSs were treated as standards, and optimization was performed to determine retention time (Rt), precursor *m/z*, product *m/z*, RF lens, and collision energies. Once all the transitions in Table S2 were optimized, they were transferred to the method file for targeted quantitation of GLSs.

### Myrosinase extraction and activity assay

Myrosinase was extracted using a previous method (39). Briefly, 100 mg rosette leaves of 5-week-old WT and mutant plants, GCs, and MCs were collected and quickly ground in liquid nitrogen with 300 μl extraction buffer (pH 7.2) consisting of 10 mM phosphate buffer, 1 mM EDTA, 3 mM DTT, and 5% glycerol. Samples were homogenized using a pestle and mortar, transferred to 1.5 ml tubes, vortexed vigorously, and centrifuged at 12,000×*g* for 15 min at 4 °C. The supernatant was collected into a separate 1.5 ml tube and kept on ice. To prepare the 1 ml reaction buffer, 33.3 mM phosphate buffer (pH 6.5), 0.2 mM sinigrin, 0.25 mM ascorbic acid, water, and 30 μl of extracted enzyme were added. Immediately after adding the extracted enzyme, the decline in absorbance at 227 nm over a 5 min period (every 30 s) was plotted. Myrosinase activity was evaluated by calculating the rate of hydrolysis of sinigrin per minute per microgram of protein. The extinction coefficient was Ɛ_227_ = 7273 M^−1^ cm^−1^ for sinigrin (73).

### Stomatal entry assay and infiltration assay

Bacterial inoculum preparation was done as previously described (44), with minor modifications. The luxCDABE-tagged *P. syringae* pv. tomato DC3000 (lux-*Pst* DC3000) (74) was provided by Dr Zhonglin Mou, University of Florida. King’s liquid media was used to grow lux-*Pst* DC3000 with kanamycin (50 mg/L) and rifamycin (25 mg/L). The bacterial inoculum was incubated at 28 °C for 24 h and harvested through centrifugation at 10,000×*g* for 10 min at room temperature. Sterilized water was added to the pellet containing the bacteria to achieve an OD of 0.2 for spray and an OD of 0.001 for injection. Spray bottles with a fine mist setting were prepared by adding 0.02% Silwet L-77. Bacteria without Silwet were used for infiltration with a needleless disposable syringe. Twelve plants were labeled for each testing group. The seventh and eighth fully developed and evenly sized leaves of each plant were marked. Sprays were applied uniformly on the abaxial side of each leaf, and infiltration was also administered to the abaxial side of the leaf. The sprayed plants were exposed to light and covered with a plastic dome to maintain high humidity for 72 h, whereas the injected plants were placed under similar conditions but without the dome. A hole puncher punched equal-sized disks of a sample (seventh and eighth leaf) and placed them in 200 μl of water with the abaxial side facing upward in a 96-well plate. The hole puncher, tweezers, and gloves were cleaned with 70% ethanol. The 96-well plate was put in the luminometer to get the readings, and the data were analyzed as log_10_ RLU/cm^2^ (Relative Luminance Unit).

## Data availability

The supporting information of all the data is available on this article's website.

## Supporting information

This article contains supporting information (60).

## Conflict of interest

The authors declare that they have no conflicts of interest with the contents of this article.
