# Supplementary material for: Cell-type–specific compartmentalization and function of the glucosinolate-myrosinase system in Arabidopsis thaliana
Source: J Biol Chem. 2026 May 27;302(7):113202. doi: 10.1016/j.jbc.2026.113202 (PMC13315440; doi:10.1016/j.jbc.2026.113202)
Supplement: Supporting Figures [file mmc1.pdf]

Single cell-type compartmentalization and function of the glucosinolate-myrosinase system in *Arabidopsis thaliana*

Shweta Chhajed[1#], Yatendra Singh[2#], Hajra Maqsood[2], Craig Dufresne[3], Wenyuan Song[4], Sixue Chen[1,2,5,6*]

[1]Department of Biology, University of Florida, Gainesville, FL, USA
[2]Department of Biology, University of Mississippi, Oxford, MS, USA
[3]Thermo Scientific Training Institute, West Palm Beach, FL, USA
[4]Department of Plant Pathology, University of Florida, Gainesville, FL, USA
[5]Plant Molecular and Cellular Biology, University of Florida, Gainesville, FL, USA
[6]Genetics Institute, University of Florida, Gainesville, FL, USA

[#]Co-first author.

[*]Correspondence:
Prof. Sixue Chen
Department of Biology, University of Mississippi, Oxford, MS, USA
Email: schen8@olemiss.edu

Table S1. List of oligonucleotide sequences used for real-time qPCR

**GC marker genes**	
SLAC1_F	GAGGGAAGAGGCGACTATGC
SLAC1_R	GGGAACAACGTTTGCCAGAC
OST1_F	ACAGGCCCCATTGTTTGTCA
OST1_R	ATTGGACCACTCACTGCTGG
**Mesophyll cell marker genes**	
PSBR_F	AGCCACGTGTCATAACCACA
PSBR_R	ACACAAGAAGAGCACCTCCG
LHCA2_F	ATCGGACCCGGACAGTCTAA
LHCA2_R	CACTTGGGTGTGAAAGCAGC
**Housekeeping/ reference gene**	
Actin2_F	GTACAACCGGTATTGTGCTGGA
Actin2_R	CAAGGTCAAGACGGAGGATG

Table S2. Selected Reaction Monitoring (SRM) transitions of identified desulfo-GLSs applied to seeds, leaves, mesophyll cells, and guard cells.

S. No.	Desulfo-GLs name	Seeds	MRM transitions	Retention time			
				Seeds	Leaves	MCP	GC
1.	3-butenylglucosinolate	3Butenyl	294.05 → 132.00	0.44	0.45	0.44	0.43
2.	2-propenyl glucosinolate	2-Prop	280.00 → 118.00	0.76	0.75	0.76	0.75
3.	3-(methylsulfinyl)-propyl	3MSOP	344.00 → 182.00	0.99	0.99	0.98	0.98
4.	4-hydroxybutyl	4OHB	312.06 → 132.00	1.15	1.44	1.44	1.44
5.	4-(methylsulfinyl)-butyl	4MSOB_Na	380.05 → 132.10	1.65	1.60	1.59	1.60
6.	5-(methylsulfinyl)-pentyl	5MSOP_Na	385.00 → 190.10	1.92	1.88	1.9	1.88
7.	4-hydroxy-indol-3-ylmethyl	4OHI3M	394.07 → 210.00	2.22	2.21	2.22	2.19
8.	6-(methylsulfinyl)-hexyl	6MSOH_Na	408.16 → 212.04	2.27	2.26	2.26	2.26
9.	3-(methylthio)-propl	3MTP	328.04 → 166.00	2.41	2.39	3.87	2.39
10.	7-(methylsulfinyl)-heptyl	7MSOH_Na	422.00 → 238.00	2.72	2.72	2.73	2.73
11.	Benzylglucosinolate	BG	330.80 → 134.20	2.82	2.82	2.81	2.80
12.	4-(methylthio)-3-butenyl	4MTB_Na	364.00 → 185.00	2.89	2.88	2.89	2.89
13.	Indol-3-ylmethyl	I3M	369.06 → 130.10	3.00	3.00	2.99	2.99
14.	8-(methylsulfinyl)-octyl	8MSOO_Na	436.00 → 274.10	3.22	3.23	3.24	3.24
15.	5-(methylthio)-pentyl	5MTP	356.07 → 147.10	3.52	3.51	3.52	3.52
16.	N-methoxy-indol-3-ylmethyl	1MTI3M	399.00 → 160.20	4.08	3.36	3.38	3.39
17.	4-methoxy-indol-3-ylmethyl	4MTI3M	399.00 → 237.00	4.08	3.36	3.38	3.39
18.	6-(methylthio)-hexyl	6MTH	370.14 → 208.08	4.22	4.2	4.22	4.21
19.	4-benxoyloxybutyl	4BOB_Na	438.11 → 242.07	4.63	4.53	6.05	4.22
20.	7-(methylthio)-heptyl	7MTH_Na	406.18 → 222.00	4.91	4.89	4.92	4.91
21.	8-(methylthio)-octyl	8MTO_Na	420.09 → 236.00	5.57	5.57	5.58	5.58

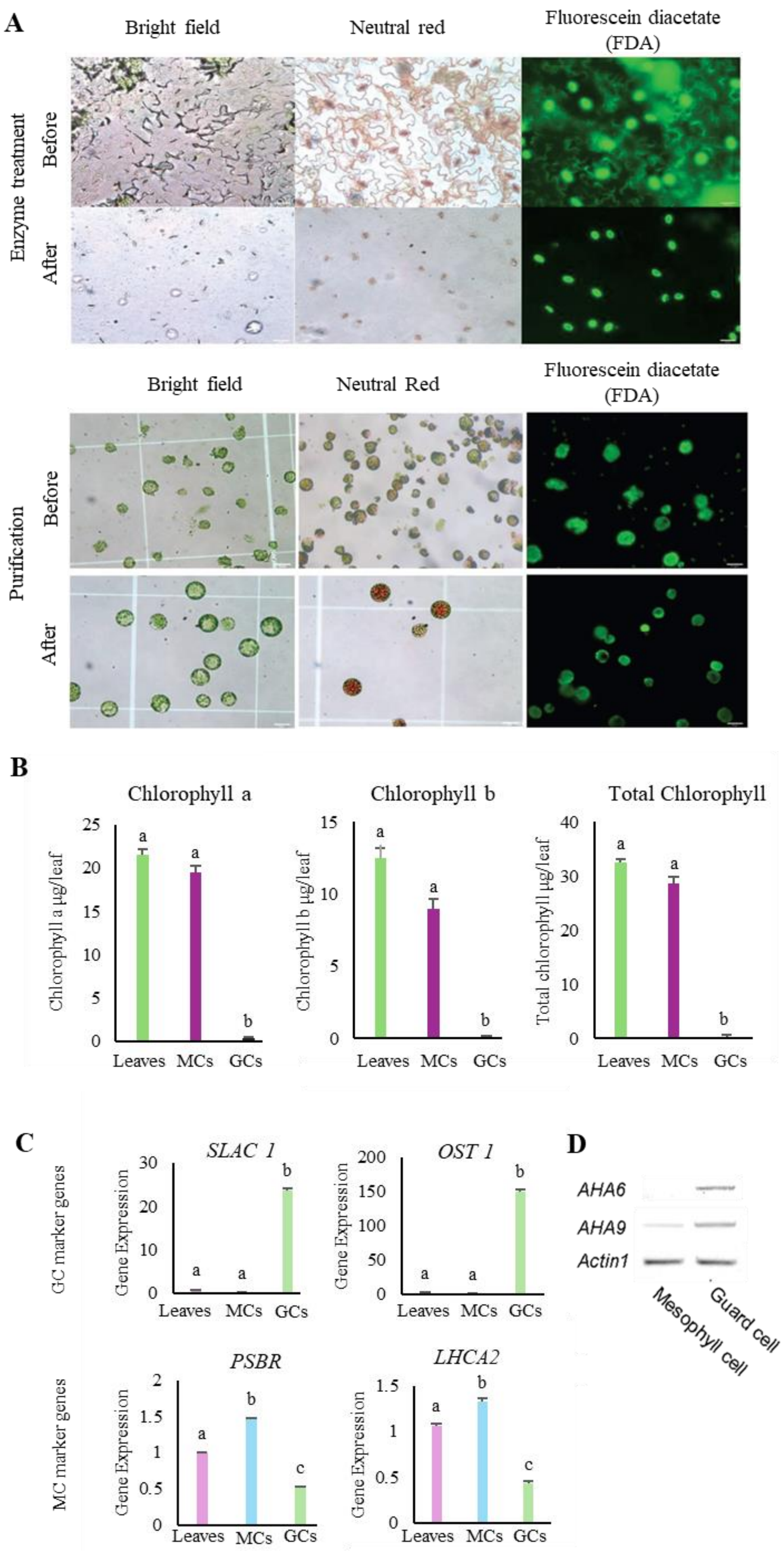

Figure S1. Purity assessment of guard cells (GCs) and mesophyll cells (MCs). (A) Viability assay of GCs and MC protoplasts. GCs in the epidermal peels were enriched after enzymatic digestion of the cell walls of epidermal cells and contaminating mesophyll cells. MC protoplasts were isolated from the same *Arabidopsis* rosette leaves used for GC preparation and purified through sucrose gradient centrifugation. Bright field, neutral red, and fluorescein diacetate (FDA) staining showing the cells are viable. (B) Chlorophyll assessment of leaves, mesophyll cells, and enriched GCs on a per leaf basis. (C) qPCR of GC and MC marker genes. The standard error of the mean was calculated from three biological replicates (from different leaves) and three technical replicates (from the same leaf). GC marker genes: *SLAC1* and *OST1*; MC marker genes: *PSBR* and *LHCA2*. Please refer to Table S1 for primer sequences used in this study. (D) Semi-quantitative RT-PCR analysis of the expression of genes encoding plasma membrane P-type H^+-ATPases *AHA6* and *AHA9* in GCs and MCs. *Actin 1*-specific primers were used to ensure that equal amounts of RNA were used, and RT reactions were equally effective for all the samples.

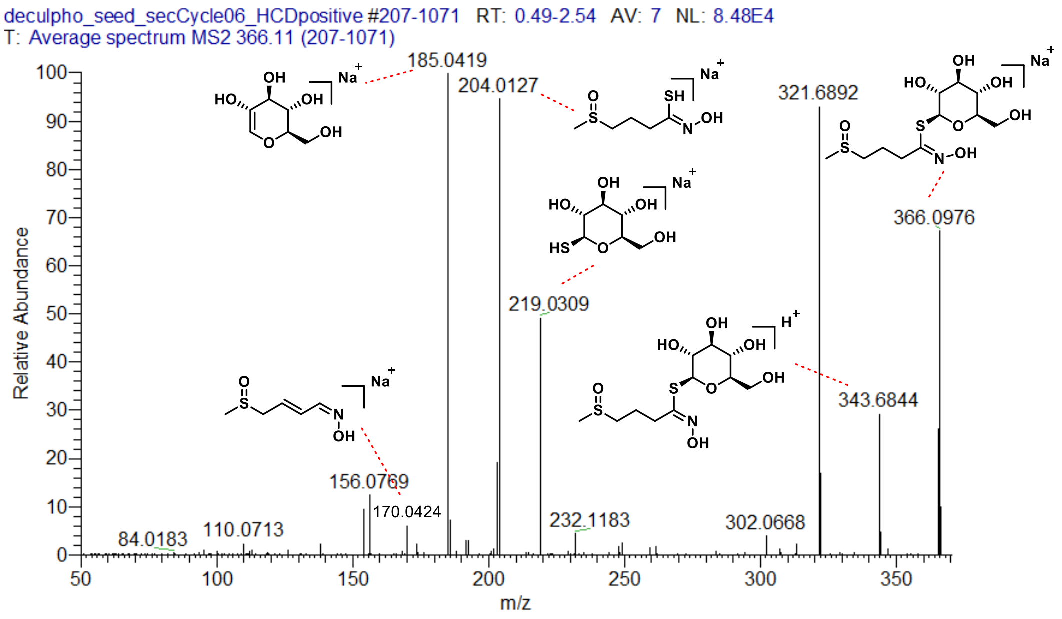

Figure S2a. Annotated MS^2 spectra of 3-(methylsulfinyl)-propyl (3MSOP-Na, *m/z* 366.0976).

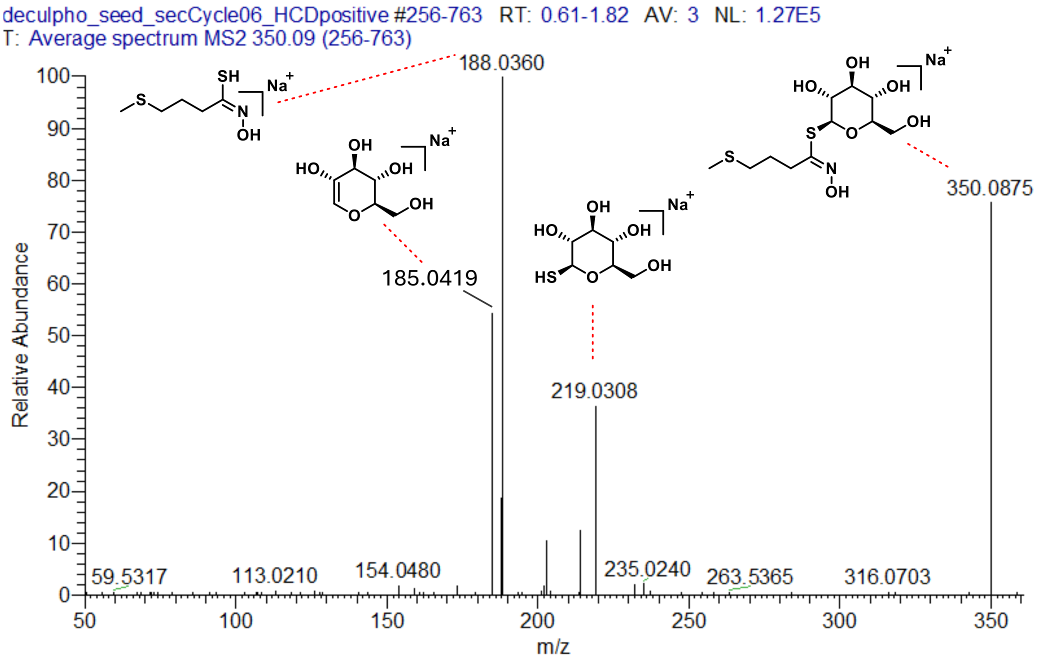

Figure S2b. Annotated MS^2 spectra of 3-(methylthio)-propyl (3MTP-Na, *m/z* 350.0875).

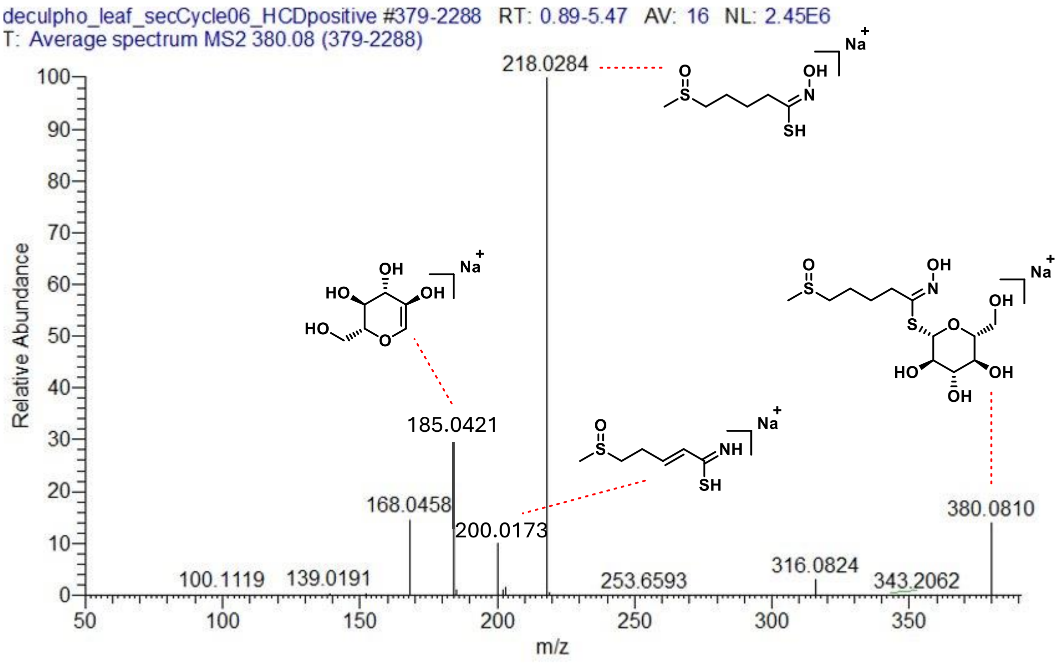

Figure S2c. Annotated MS^2 spectra of 4-(methylsulfinyl)-butyl (4MSOB-Na, *m/z* 380.0810).

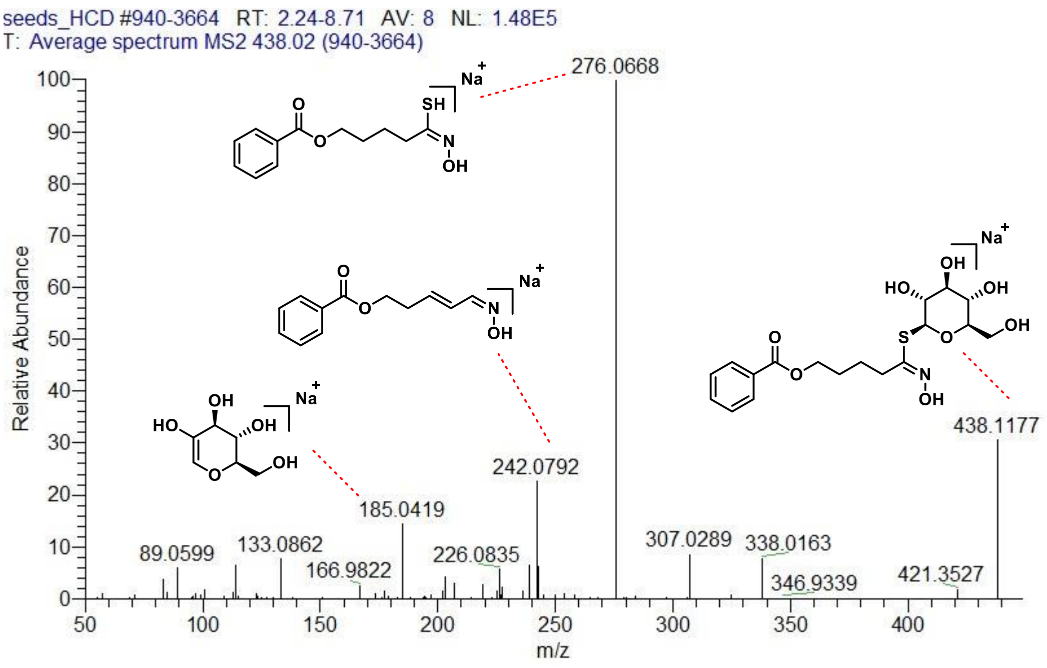

Figure S2d. Annotated MS^2 spectra of 4-benxoyloxybutyl (4BOB-Na, *m/z* 438.117).

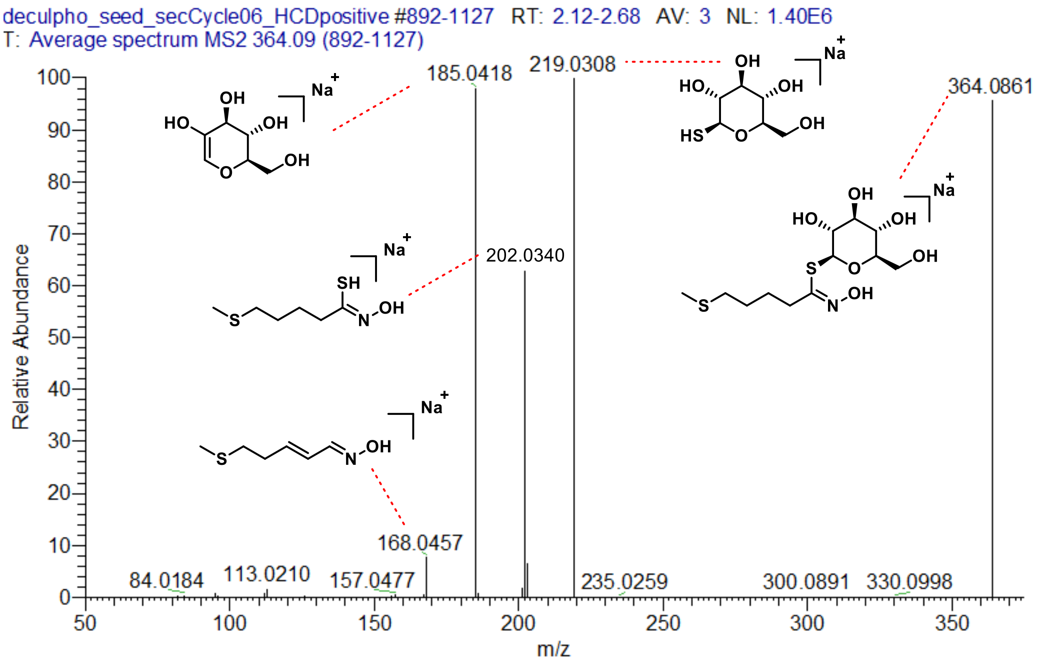

Figure S2e. Annotated MS^2 spectra of 4-(methylthio)-3-butenyl (4MTB-Na, *m/z* 364.0861).

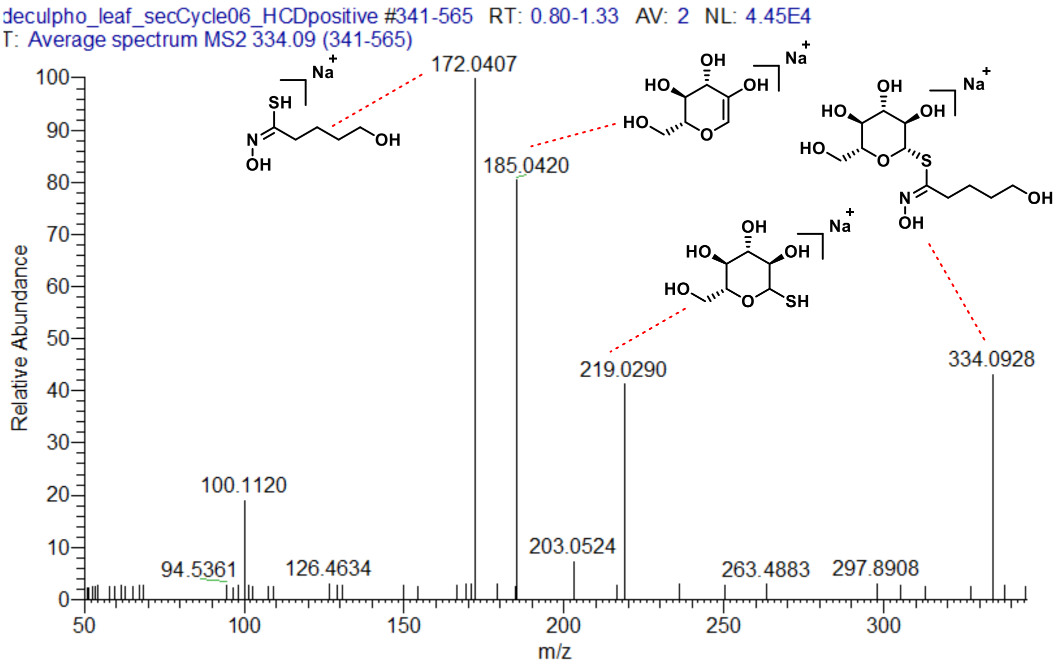

Figure S2f. Annotated MS^2 spectra of 4-hydroxybutyl (4OHB-Na, *m/z* 334.0928).

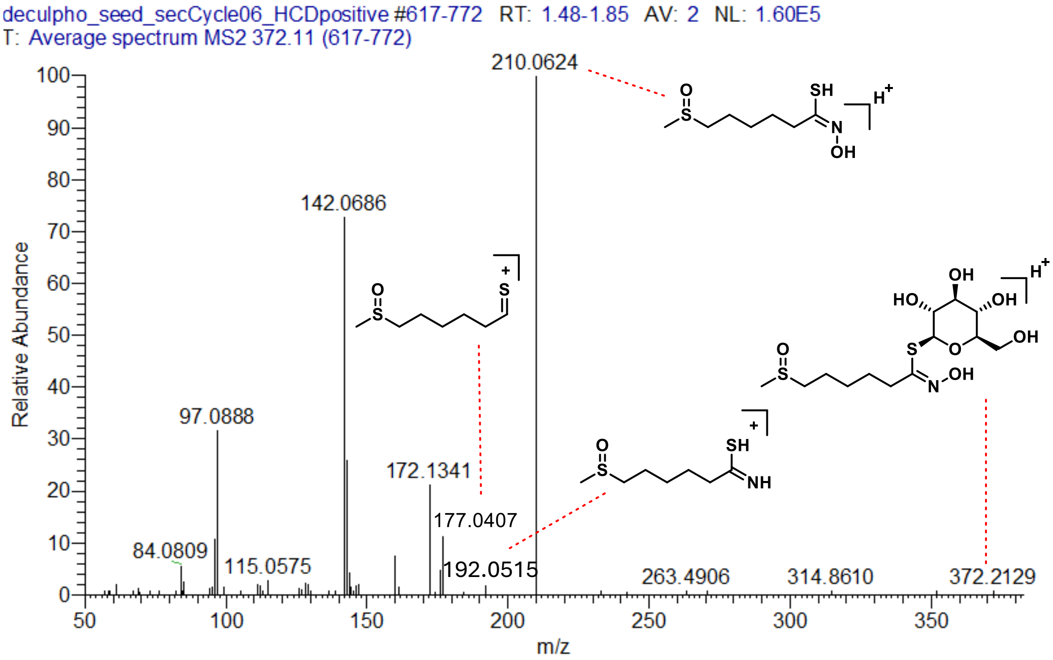

Figure S2g. Annotated MS² spectra of 5-(methylsulfinyl)-pentyl (5MSOP-Na, *m/z* 372.2129).

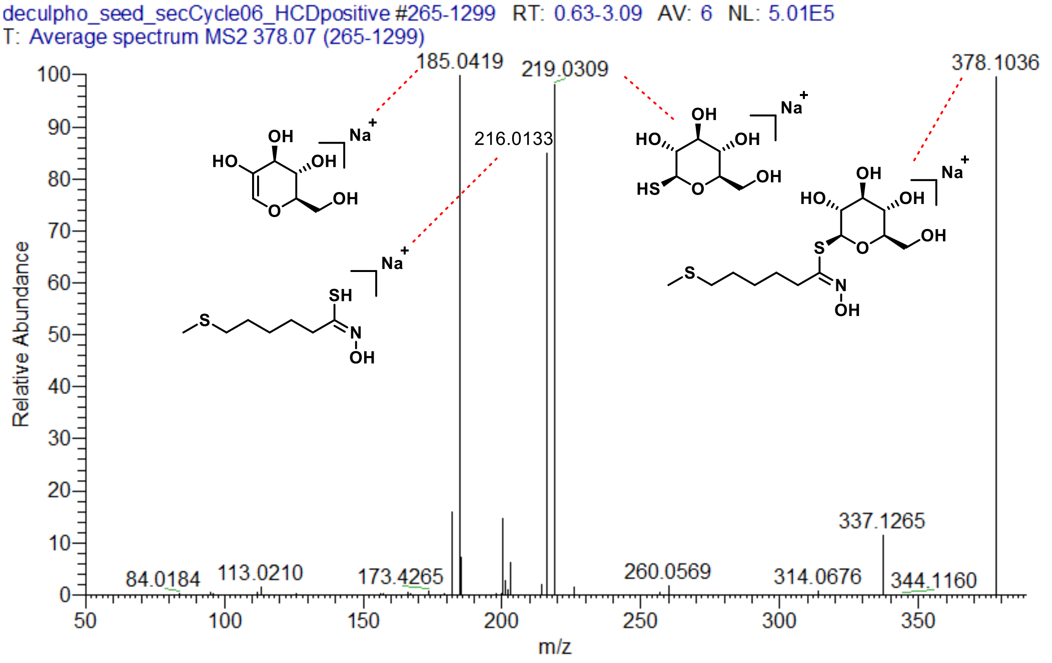

Figure S2h. Annotated MS² spectra of 5-(methylthio)-pentyl (5MTP-Na, *m/z* 378.1036).

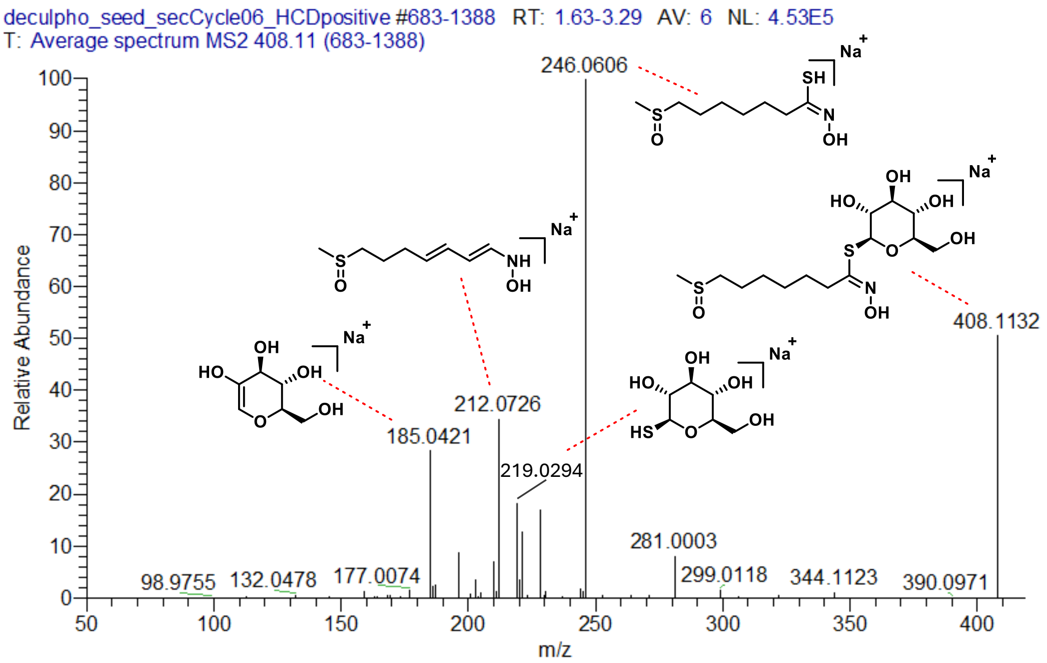

Figure S2i. Annotated MS[2] spectra of 6-(methylsulfinyl)-hexyl (6MSOH-Na, *m/z* 408.1132).

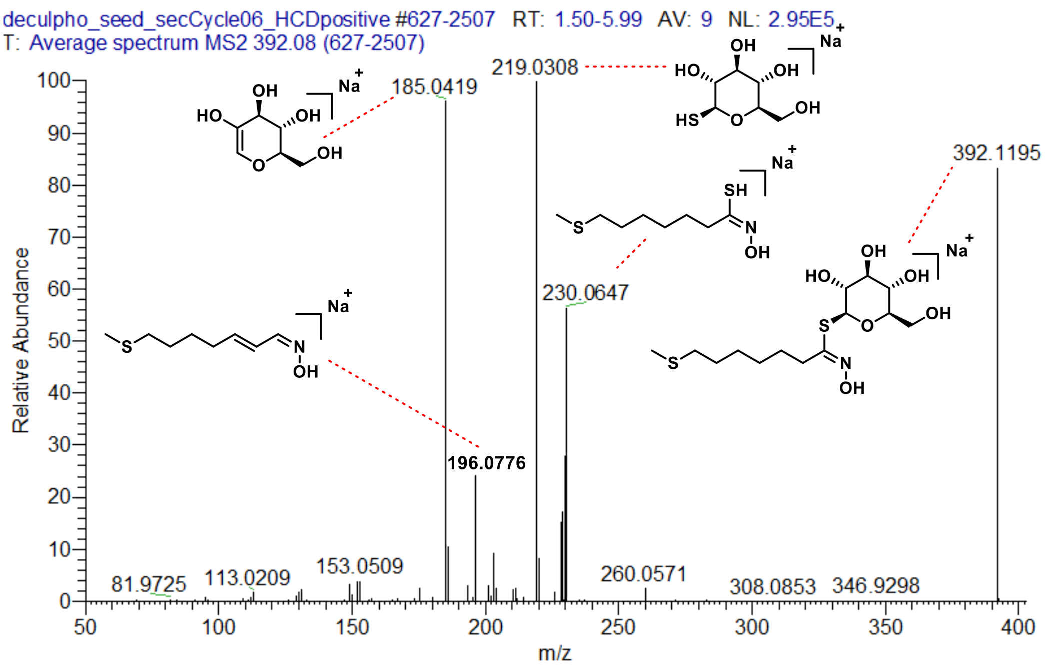

Figure S2j. MS[2] spectra of 6-(methylthio)-hexyl (6MTH-Na, *m/z* 392.1195).

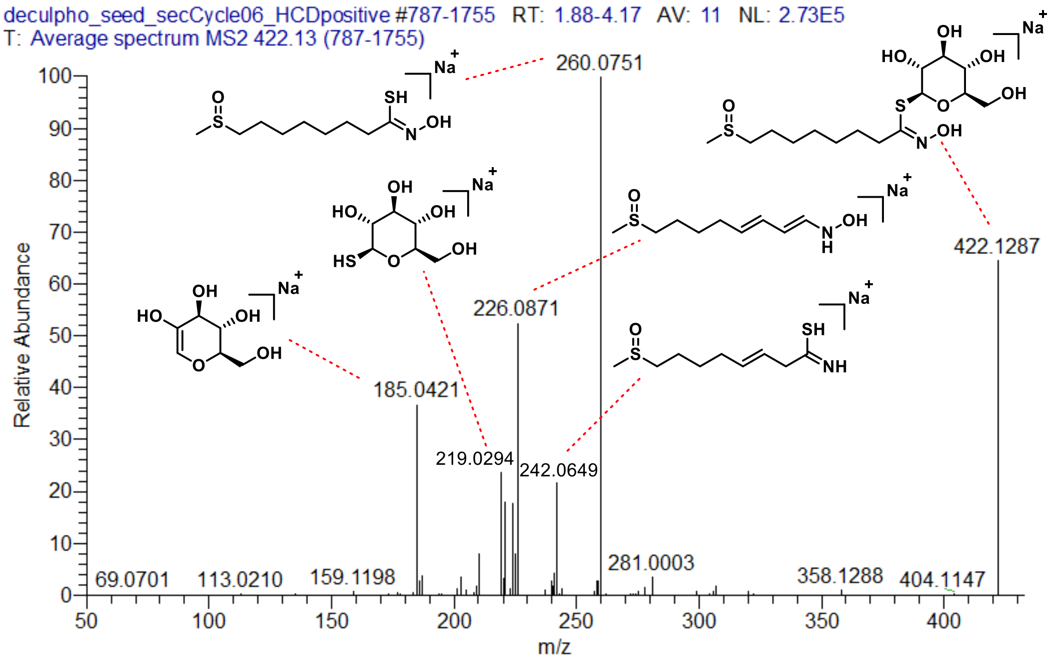

Figure S2k. Annotated MS² spectra of 7-(methylsulfinyl)-heptyl (7MSOH-Na, *m/z* 422.1287).

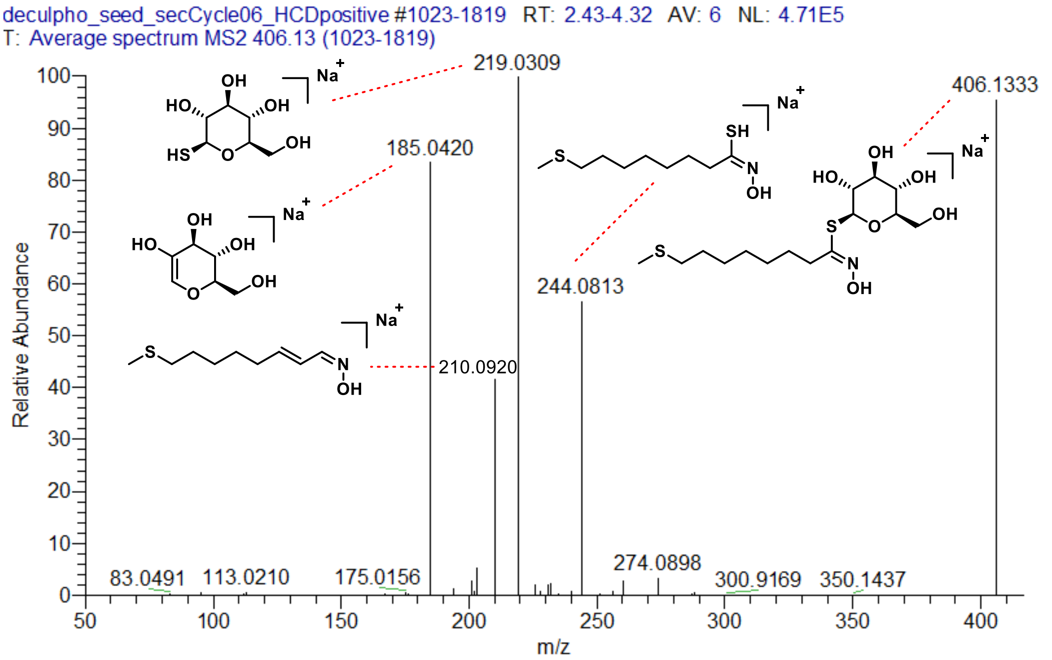

Figure S2l. Annotated MS² spectra of 7-(methylthio)-heptyl (7MTH-Na, *m/z* 406.1333).

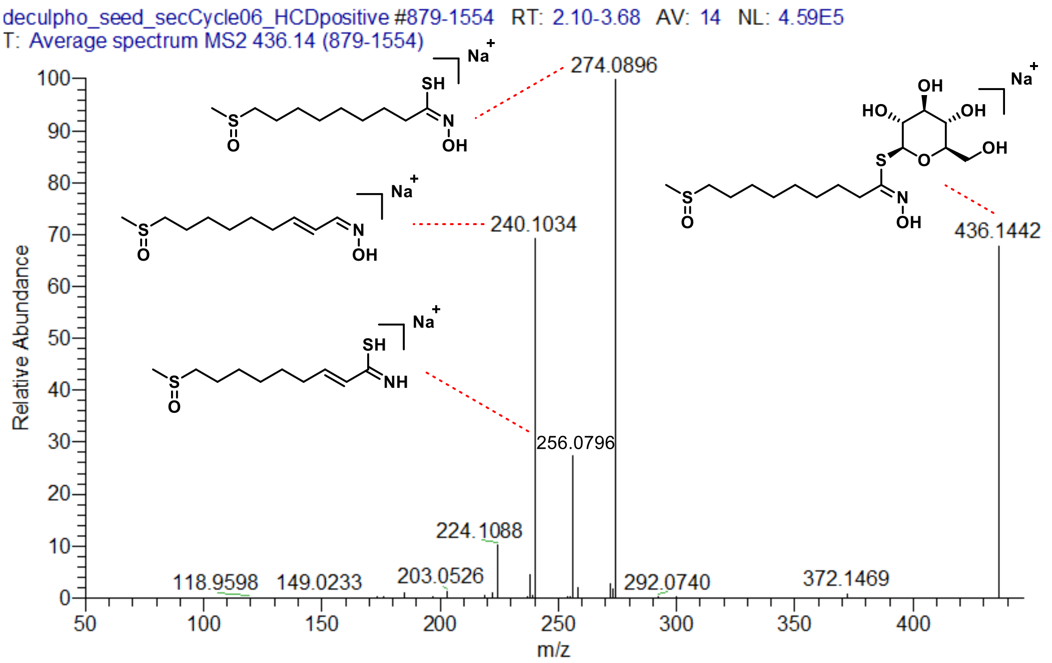

Figure S2m. Annotated MS^2 spectra of 8-(methylsulfinyl)-octyl (8MSOO-Na, *m/z* 436.1442).

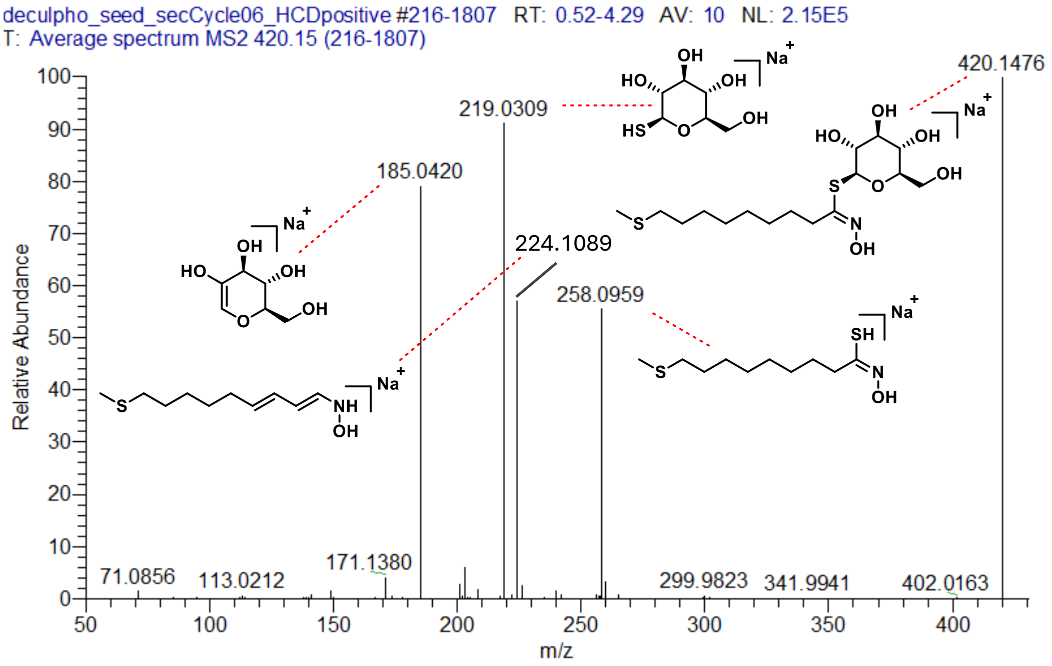

Figure S2n. Annotated MS^2 spectra of 8-(methylthio)-octyl (8MTO-Na, *m/z* 420.1476).

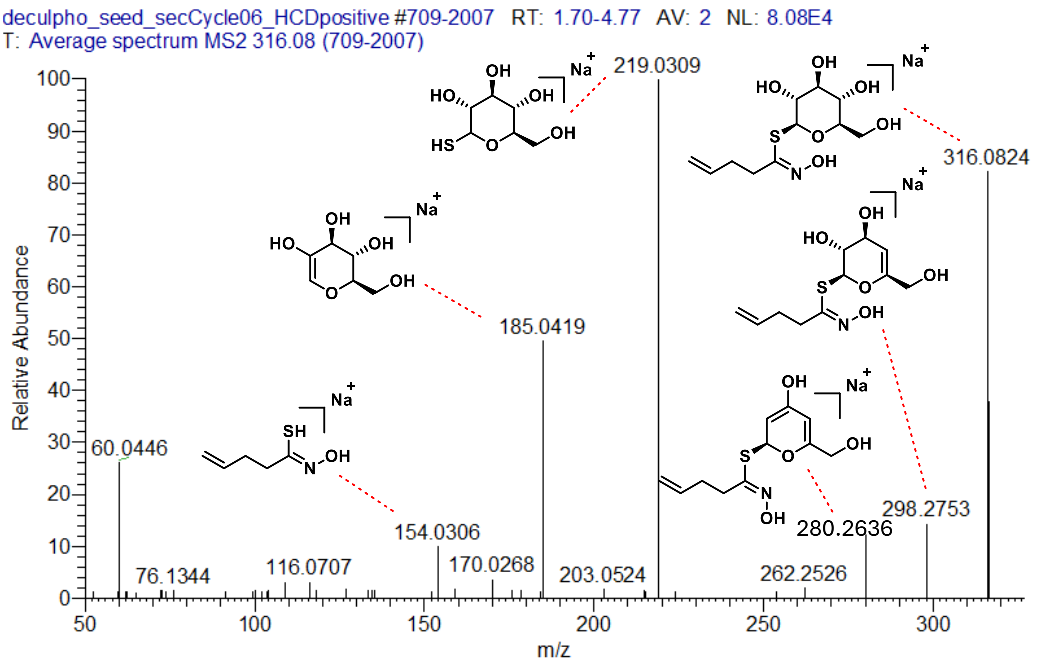

Figure S2o. Annotated MS^2 spectra of 3-butenylglucosinolate (3Butenyl-Na, *m/z* 316.0824).

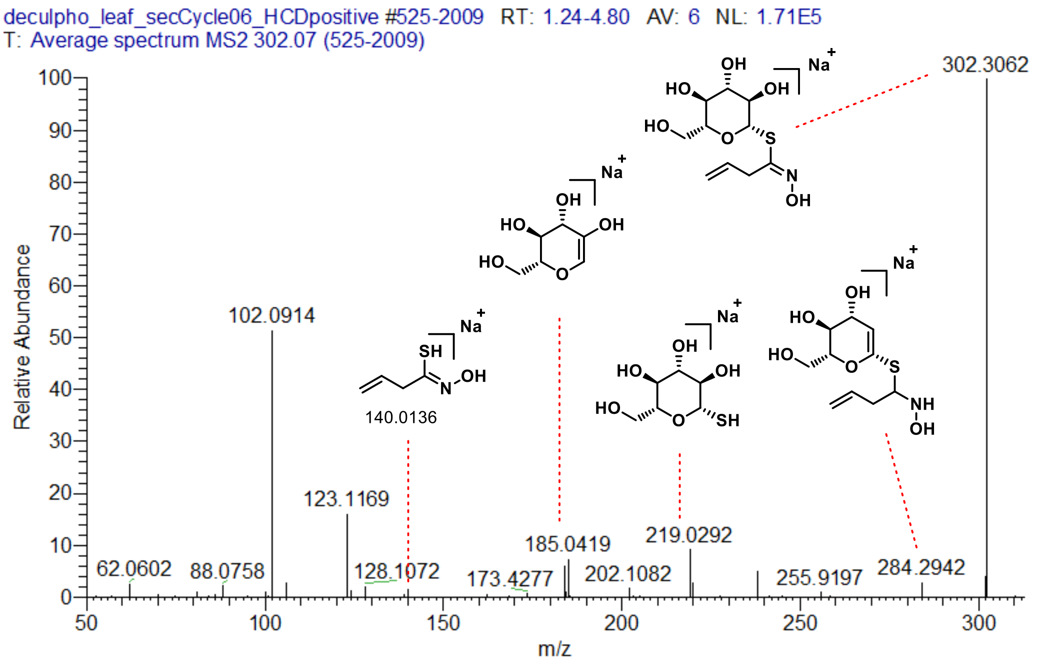

Figure S2p. Annotated MS^2 spectra of 2-Propenyl glucosinolate (3Prop-Na, *m/z* 302.3062).

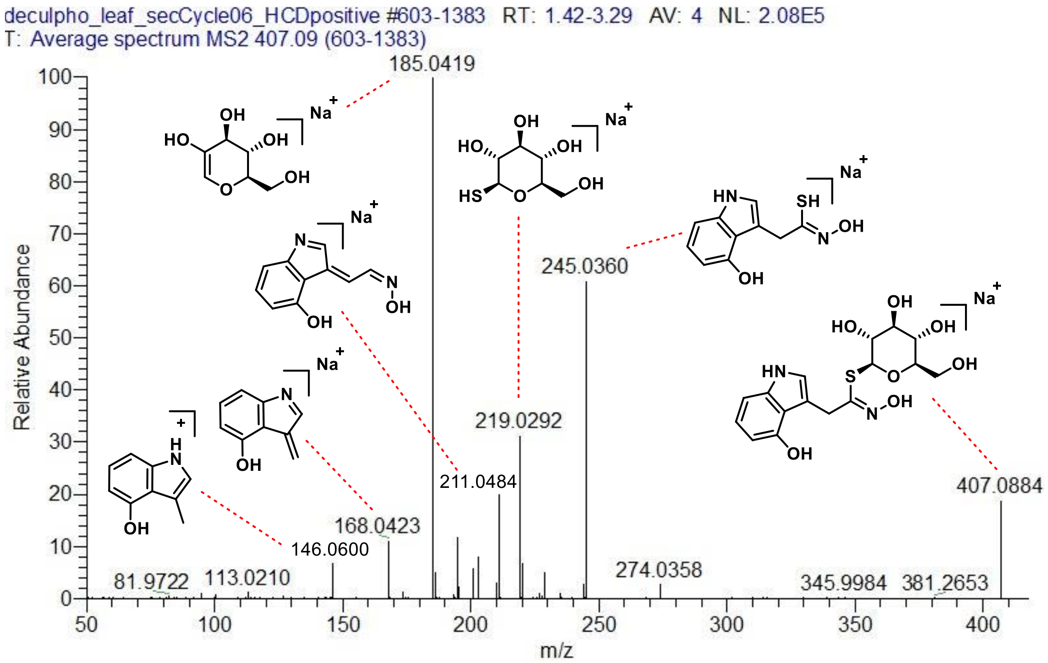

Figure S2q. Annotated MS^2 spectra of 4-hydroxy-indol-3-ylmethyl (4OHI3M+Na, *m/z* 407.0884).

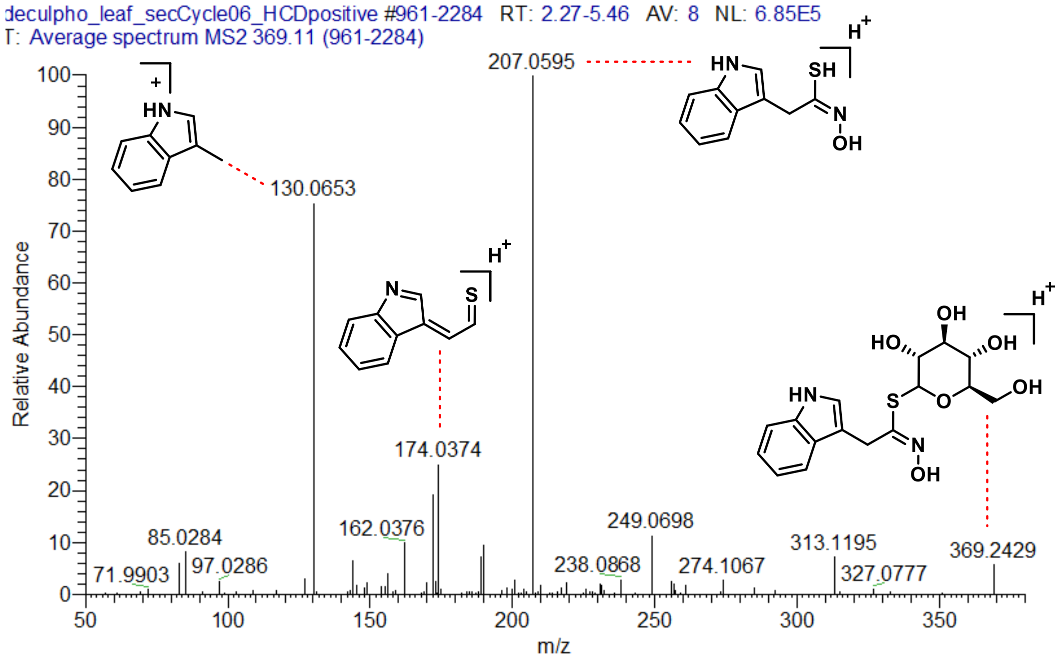

Figure S2r. Annotated MS^2 spectra of indol-3-ylmethyl (I3M-H, *m/z* 369.2429).

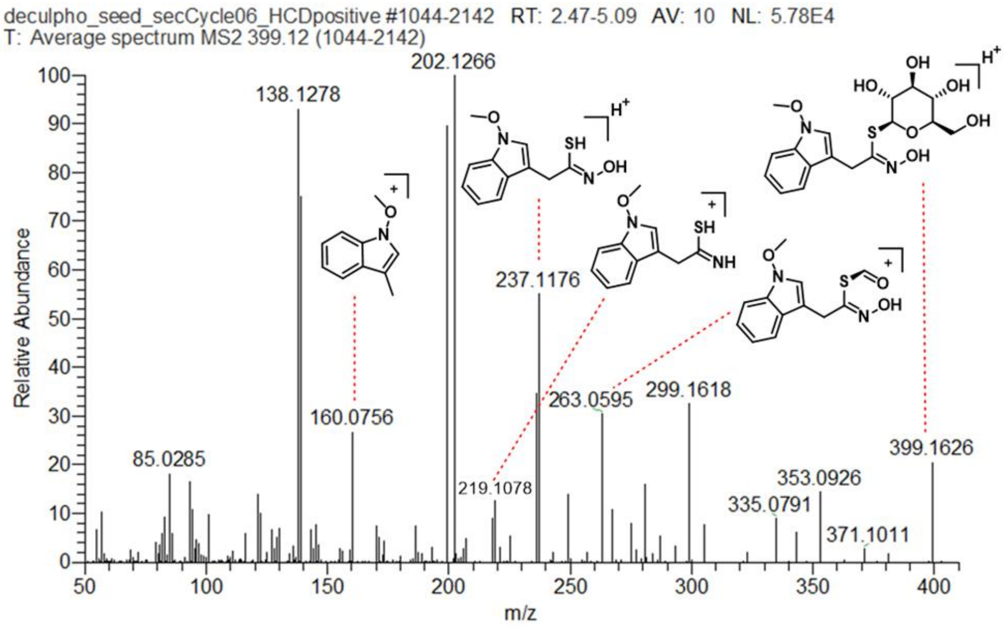

Figure S2s. Annotated MS^2 spectra of N-methoxy-indol-3-ylmethyl (1MTI3M+H, *m/z* 399.1626).

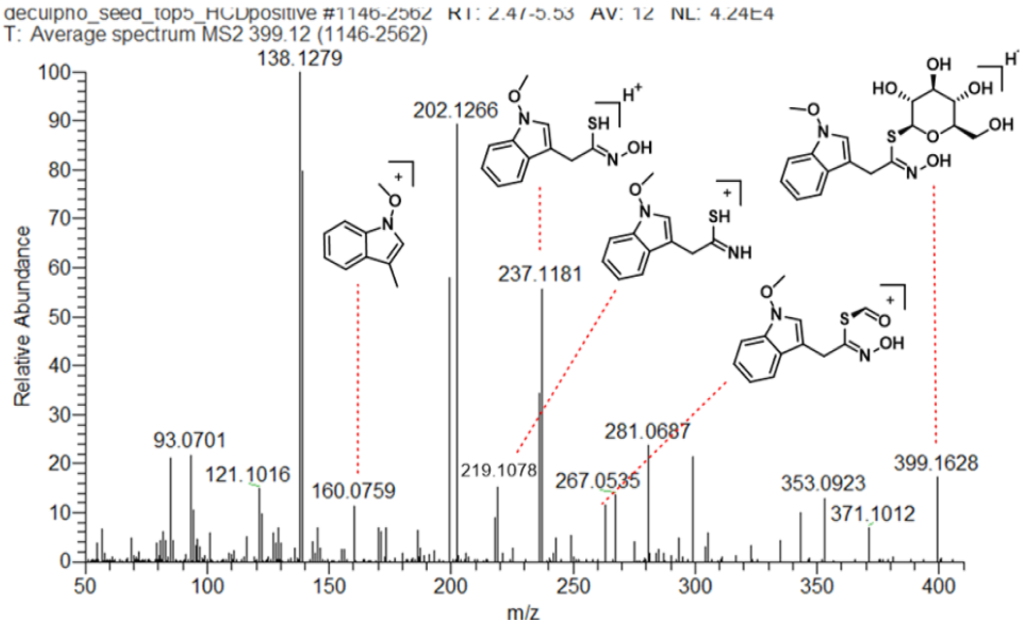

Figure S2t. Annotated MS^2 spectra of 4-methoxy-indol-3-ylmethyl (4MTI3M+H, *m/z* 399.1626).

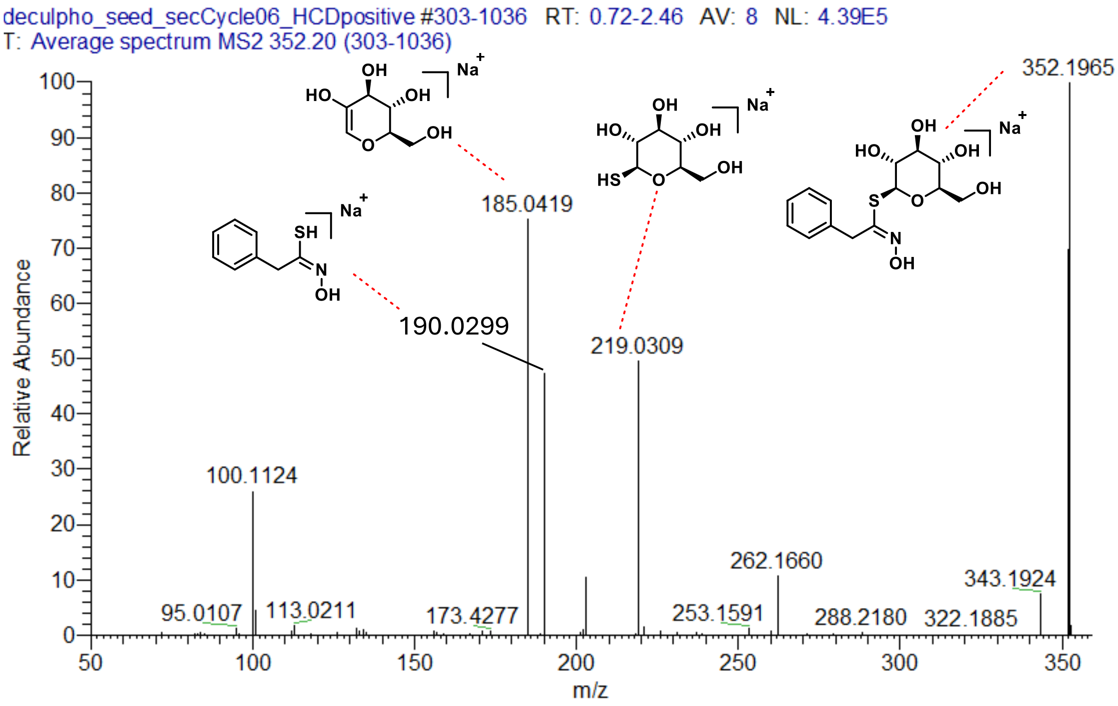

Figure S2u. Annotated MS^2 spectra of benzylglucosinolate (BG-Na, *m/z* 352.1965).

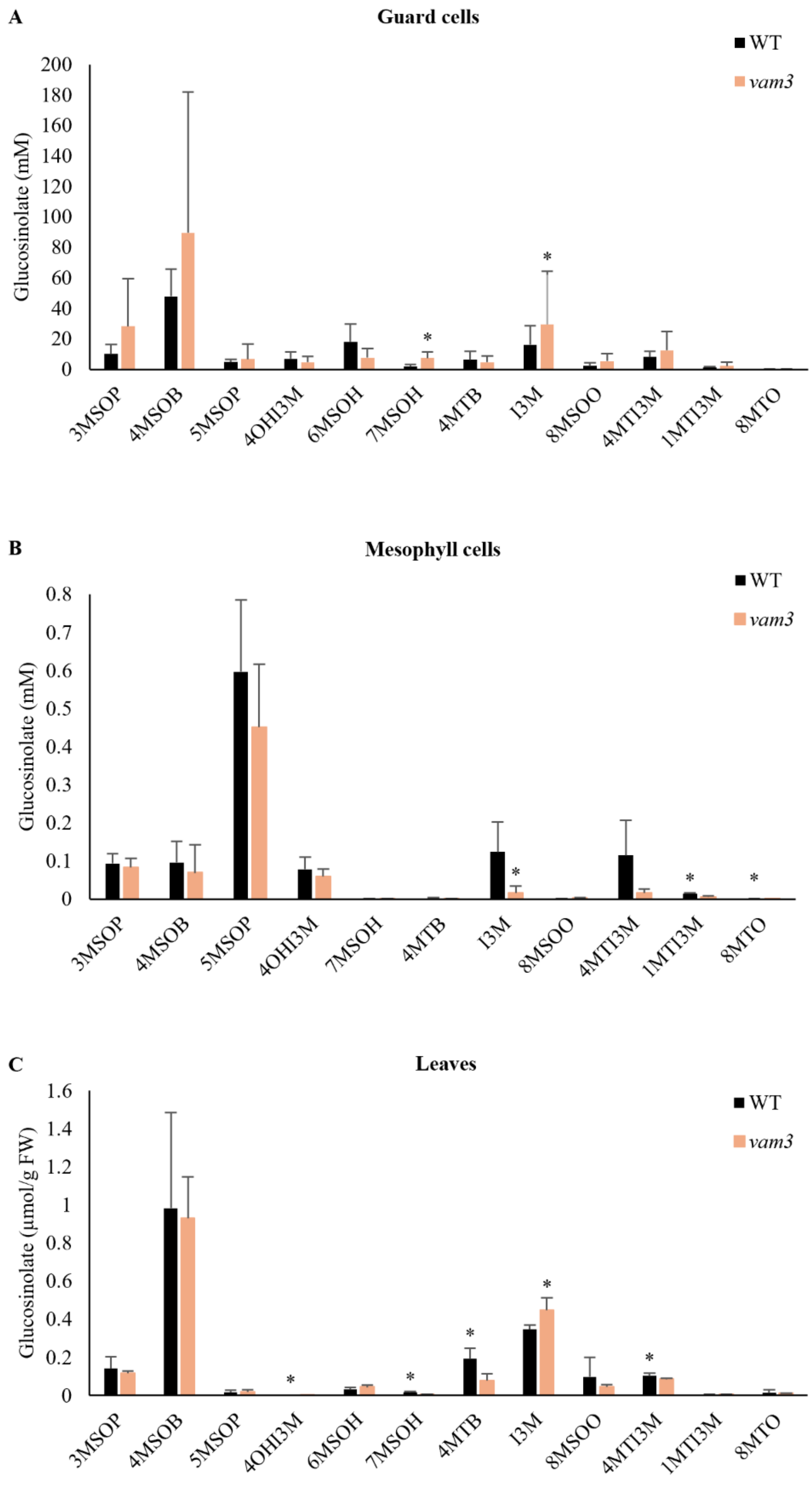

Figure S3. Glucosinolate (GLS) profiles in different cell types of *A. thaliana*. (A) Quantitative GLS profiles in guard cells (GCs), (B) Mesophyll cells (MCs) of WT and *vam3*, (C) Glucosinolate concentrations in leaves of WT and *vam3*. Data show mean (± SD) from three biological replicates, and pairwise t-test was used for statistical analysis (* indicating P < 0.05).

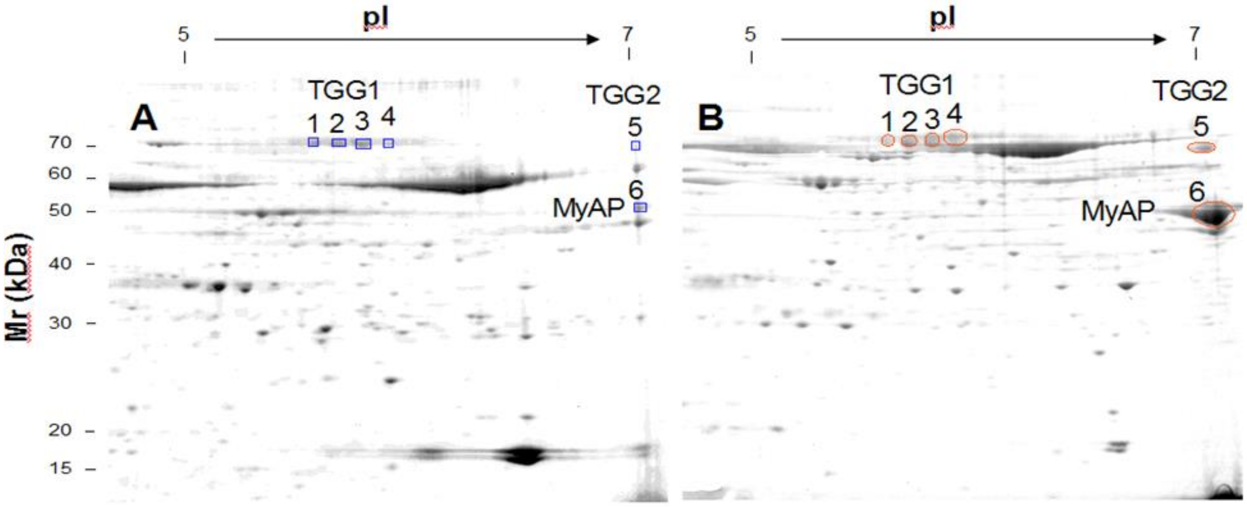

Figure S4. 2D gel maps of vacuolar proteins from *Arabidopsis* rosette leaves. (A) Vacuoles from 16-day-old leaves; (B) Vacuoles from 6-week-old rosette leaves. The labeled protein spots were unambiguously identified using LC-MS/MS and database searching, with spots 1-4 identified as myrosinase TGG1, spot 5 as TGG2, and spot 6 as MyAP1 (60).

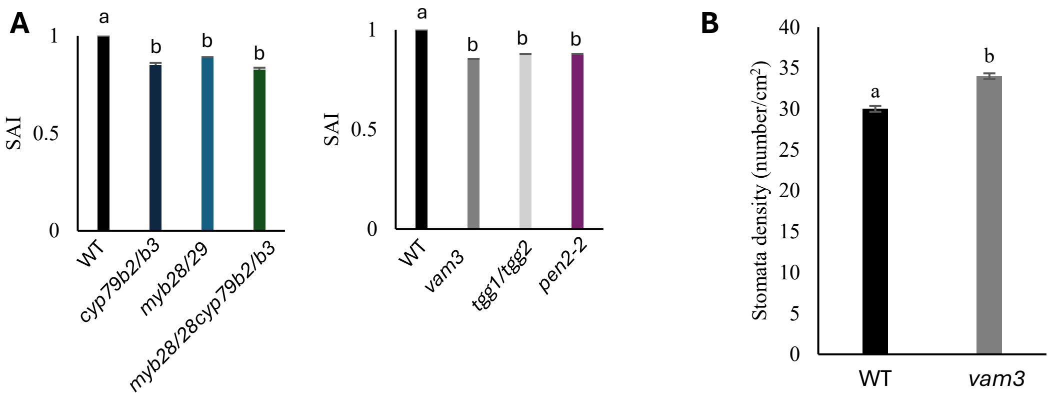

Figure S5. Stomata aperture index (SAI) and stomata density of different glucosinolate and myrosinase mutants. (A) SAI calculated using the ratio between the width and the length of stomata; (B) Stomata density (number of stomata per cm^2) of wild type (WT) and the mutant *vam3* with high TGG1 and TGG2 abundance. Different letters indicate a statistical difference between the comparisons.

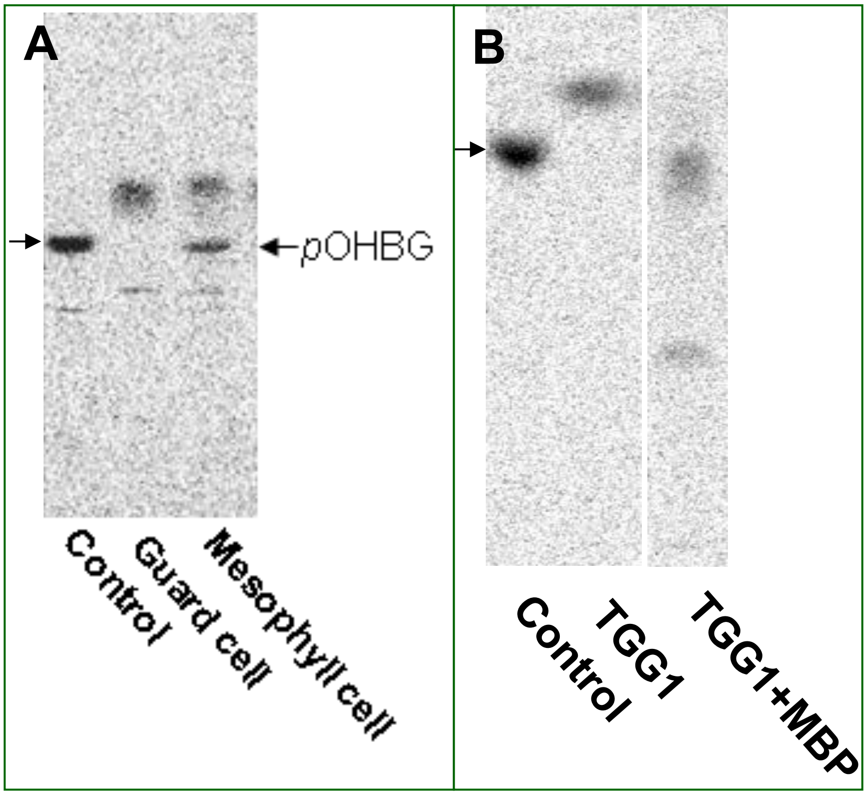

Figure S6. Comparison of myrosinase activities in GCs and MCs of *Arabidopsis* leaves, and the effect of a myrosinase binding protein (MBP2). (A) Thin-layer chromatography analysis of *p*-hydroxybenzylglucosinolate (*p*-OHBG) (arrow) degradation profile by TGG1; (B) *p*-OHBG (arrow) degradation profile by GC TGG1, and TGG1 incubated with MBP2.

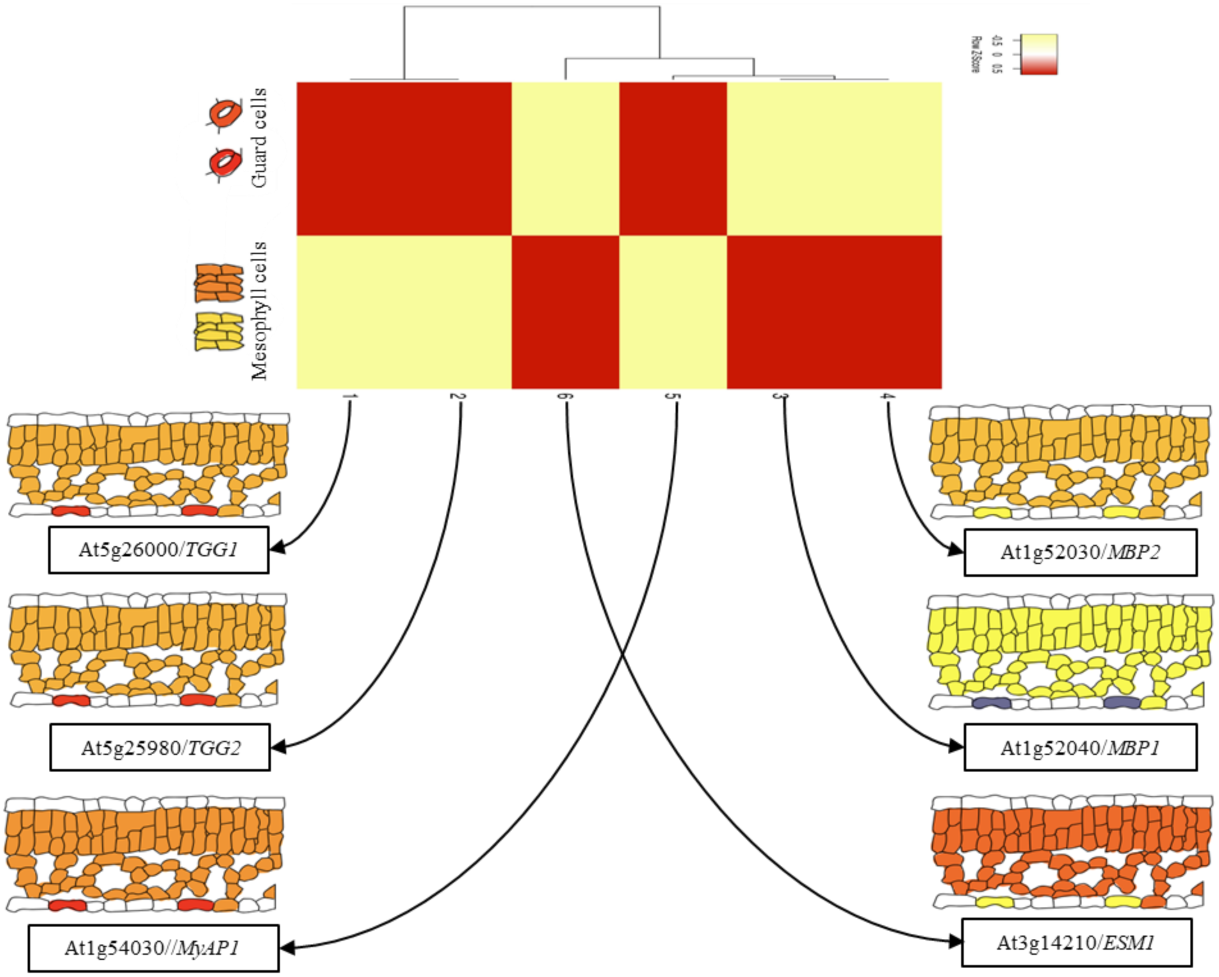

Figure S7. Expression levels of myrosinase genes *TGG1* and *TGG2*, myrosinase associate protein 1 (*MyAP1*), myrosinase binding protein (*MBP1*), *MBP2*, and epithiospecifier modifier 1 (*ESM1*) in *Arabidopsis* GCs and MCs. Genes retrieved from the TAIR database (https://www.*Arabidopsis*.org/) and subjected to E-plant (https://bar.utoronto.ca/eplant/) to construct a heat map and cell-type-specific expression patterns. Red to yellow color scales denote the high to low expression.
